# Multiple levels of contextual influence on action-based timing behavior and cortical activation

**DOI:** 10.1038/s41598-023-33780-1

**Published:** 2023-05-02

**Authors:** Ali Rahimpour Jounghani, Pradyumna Lanka, Luca Pollonini, Shannon Proksch, Ramesh Balasubramaniam, Heather Bortfeld

**Affiliations:** 1 Department of Psychiatry and Behavioral Sciences, C-Brain Lab, Stanford University School of Medicine, Stanford, CA USA; 2 Psychological Sciences & Cognitive and Information Sciences, University of California, Merced, CA USA; 3 Department of Engineering Technology, Electrical and Computer Engineering, and Biomedical Engineering, University of Houston, Houston, TX USA; 4 Basque Center On Cognition, Brain and Language, San Sebastian, Spain; 5 Department of Psychology, Augustana University, Sioux Falls, SD USA; 6 Cognitive & Information Sciences, University of California, 5200 N Lake Rd, School of Social Sciences, Humanities and Arts, Room SSM 247B, Merced, CA 95343 USA

**Keywords:** Psychology, Human behaviour

## Abstract

Procedures used to elicit both behavioral and neurophysiological data to address a particular cognitive question can impact the nature of the data collected. We used functional near-infrared spectroscopy (fNIRS) to assess performance of a modified finger tapping task in which participants performed synchronized or syncopated tapping relative to a metronomic tone. Both versions of the tapping task included a pacing phase (tapping with the tone) followed by a continuation phase (tapping without the tone). Both behavioral and brain-based findings revealed two distinct timing mechanisms underlying the two forms of tapping. Here we investigate the impact of an additional—and extremely subtle—manipulation of the study’s experimental design. We measured responses in 23 healthy adults as they performed the two versions of the finger-tapping tasks either *blocked* by tapping type or *alternating* from one to the other type during the course of the experiment. As in our previous study, behavioral tapping indices and cortical hemodynamics were monitored, allowing us to compare results across the two study designs. Consistent with previous findings, results reflected distinct, context-dependent parameters of the tapping. Moreover, our results demonstrated a significant impact of study design on rhythmic entrainment in the presence/absence of auditory stimuli. Tapping accuracy and hemodynamic responsivity collectively indicate that the block design context is preferable for studying action-based timing behavior.

## Introduction

Timing and coordination in human behavior can be described as a function of motor and perceptual interactions within a larger complex system^1^. The behavior of such a system can be affected by changes in the timing of both exogenous, observed stimuli and endogenous processes. Thus, it is critical to characterize an appropriate contextual approach to study human timing abilities. Sensorimotor synchronization is a scientific approach to investigating behavioral timing entrainment in humans, a process that fundamentally underlies more complex rhythmic behaviors (e.g., dancing). Finger-tapping is a common sensorimotor synchronization task used in the exploration of timing control of rhythmic entrainment (i.e., establishing and maintaining a stable temporal relationship between external periodic inputs and endogenous rhythmic process)^2,3^. Finger-tapping studies assess how participants use their mental timing system in a manner that is independent of other motor behavior or feedback mechanisms^4–6^. Two leading derivatives of the sensorimotor synchronization approach are synchronization-continuation and syncopation-continuation tasks (tapping on the beat, or off the beat respectively)^7,8^. In these approaches, participants are initially entrained to an external stimulus, such as an auditory tone. When the stimulus train is extinguished, they are asked to maintain the behavior (tapping on or off the beat) based on their own, internal timekeeping mechanism.

Two stable modes of human behavioral coordination to external signals are synchrony and syncopation (also referred to as anti-synchrony). Synchronized tapping to an external pacing signal generally requires little preparation and self-monitoring^9,10^. By contrast, syncopated tapping requires substantial monitoring of the perception–action cycle across time^10^. Participants may also be asked to continue tapping after the cessation of the externally presented stimulus, which allows researchers to contrast their accuracy in pacing to the external stimulus and continuing without it. Such a “continuation phase” depends on an internal mental representation of the duration of the intervals between the external stimulus, and thus can provide insights into specific aspects of timing behavior and endogenous timing processes.

The ability to accurately and precisely perform temporally accurate behavior is critical to various real-world skills^11^ and thus the topic of extensive research. Different task-specific timing parameters can be manipulated to further target specific neural and/or cognitive mechanisms underlying the behavior. Behavioral performance and the neural activity depend on several timing parameters, including duration of timing movement. Other factors that have been shown to impact human motor behavior include: individual experience (musician vs. non-musician)^12^, interval ratio (metrical vs. non-metrical) and rhythmic complexity of a stimulus^13,14^, an individual’s rate of movement^15^, the overall number of stimuli^16^, specific kinematics of the movement (e.g., velocity of acceleration)^17^, switch of effector^18^, movement trajectory^19^, engagement of a dominant versus non-dominant limb^20^, and unimanual versus bimanual movement^18^. In short, coordination in human behavior is the product of multiple interactions within a larger complex dynamical system. It follows that subtle decisions in the initial design of timing tasks may impact corresponding behavioral and neural processes, and the conclusions that can be drawn from them.

### Study designs in timing research

Given the intricacies of timing behavior and the wide variety of contexts in which humans make use of different timing mechanisms, it is critical that experimental tasks are designed to ensure that stable characteristics of timing behavior are observed. To this end, research on motor control and movement generally includes regular alternations between task conditions to minimize behavioral carry-over—and thus confounds—across trials^21^. By contrast, fMRI research on motor control and timing more often has relied on the traditional block design, with the aim of improving signal to noise ratio in neural data across conditions^22^. Here we describe a systematic comparison of two experimental designs—blocked and alternating—in the context of an investigation of behavioral and neural correlates of timing.

In a previous study (see^8^), we asked participants to perform synchronization-continuation and syncopation-continuation finger-tapping tasks. These synchronized and syncopated tapping trials were presented in a block design, so participants did not have to switch back and forth between the two types of tapping. Participants were asked to pace their tapping relative to the auditory tone and then continue tapping when the tone ceased. Trials were presented in a blocked format with order of tapping mode counterbalanced across participants. This a standard block design and in this context, rather than switching back and forth between the two modes of tapping, participants performed all the trials required for one mode of tapping followed by all the trials for the other mode. Over the course of the study, we collected both behavioral and neurophysiological measures, the latter using functional near-infrared spectroscopy (fNIRS). FNIRS provides detailed information about moment-to-moment changes in both oxygenated [oxy-Hb] and deoxygenated [deoxy-Hb] hemoglobin, albeit with activity constrained to the cortex (and thus not including subcortical structures).

Our own and others’ research using the design outlined above has revealed differential recruitment of sensorimotor cortex (S1/ M1), supplementary motor area (SMA), premotor cortex (PMC), inferior parietal cortex, basal ganglia, and cerebellum, all depending on the mode and phase of tapping being performed^23,24^. Our results showed that hemodynamic changes were directly related to the complexity of the tapping task, with a broader cortical network recruited during the syncopation-continuation condition compared to other three conditions (synchronized pacing, synchronized continuation, or syncopated pacing). The block design context activated a network compatible with the motor-related timing network (M1 and SMA) in both the synchronized and syncopated tapping modes. Additional activity was observed during the syncopated relative to the synchronized mode of tapping in central, frontal, and parietal areas, a finding that is consistent with the increased memory and attention processes this type of tapping engages, in addition to its overall increase in cognitive control demands^8^.

In contrast, an alternating design describes a study design whereby consecutive trials alternate between experimental conditions. In this design, participants switch between tapping modes for each consecutive set of trials while performing both pacing and continuation phases within each trial set^19,25,26^. While the two tapping modes—synchronous and syncopated—can serve as stable attractors for rhythmic movement, the syncopated mode is generally less stable^8^. Moreover, with increased task demands—including the frequent switching between tapping modes and the corresponding reduction in time available to develop an internal timing representation^27^—the alternating design should result in less accurate tapping and greater neural engagement than the block design, particularly when syncopated and/or continuation is involved.

### Current study

The current study was designed to determine whether a design manipulation—blocking tapping modes or alternating between modes—would impact behavioral and neural measures of rhythmic entrainment. We employed the same finger-tapping task we used in our previous study (Rahimpour et al., 2020). This task included the two tapping modes (synchronized vs. syncopated) and the two maintenance phases (pacing with and continuing without an auditory). The critical manipulation was that half of the participants performed the task with trials blocked by tapping mode, while trials for the other half alternated between modes. Thus, in addition to the within-participant experimental conditions we tested in our previous study—synchronized pacing, synchronized continuation, syncopated pacing, and syncopated continuation—participants here performed the tapping in one of two high-level design contexts (blocked or alternating).

We hypothesized that participants who alternated between two distinct modes of tapping would experience increased task demands, and thus perform worse relative to those performing the two modes of tapping in blocks. Such a finding would demonstrate that, not only does the relative complexity of exogenous and endogenous aspects of a timing task matter to performance, but also that the contextual complexity in which the task is embedded impacts performance.

As in our previous study, we tracked tapping accuracy using a behavioral measurement (MakeyMakey™ kit) coupled with a measure of cortical hemodynamics (fNIRS). Compared to fMRI, fNIRS provides fine-grained temporal information and a more complete view of cortical hemodynamic activity, providing information about concentration changes of both [oxy-Hb] and [deoxy-Hb] hemoglobin^28^. Critically, fNIRS allows for considerations of ecological validity as measurements can be taken while participants are sitting upright and moving more naturally than is the case with fMRI. Moreover, the MakeyMakey™ kit allows for participants to tap freely without experiencing the resistance often associated with button pressing.

To test our hypothesis that the high-level context in which a task is embedded interacts with specific low-level task parameters, we compared participants’ performance accuracy and the amount and breadth of their cortical engagement across the two study designs. Specifically, we measured accuracy using both an asynchrony index and comparisons of inter-response interval (IRI) across the different task conditions and in design contexts. We also compared relative changes in participants’ cortical hemodynamics while they performed the task. This allowed us to probe the neural representation and continuity of timing behavior across two different tapping modes (synchronization and syncopation), two different maintenance phrases (pacing and continuation), and two different design contexts (blocked and alternating). We predicted that the finger-tapping task would produce markedly different behavioral and cortical outcomes depending on the specific task parameters and the context in which they were embedded.

## Results

Our focus is on the impact of the two study designs on the behavioral and hemodynamic measures of the finger-tapping task, above and beyond the contextual influences of mode and phase of the maintenance paradigm. We first report and compare the behavioral results from the IRI and mean asynchrony indices, followed by a comparison of the channel maps for each condition across the block and alternating study designs.

### Behavior

#### Mean asynchrony

Our estimated multiple regression model was calculated to predict mean asynchrony based on mode, phase, and study design. A significant effect of the independent variables on the mean synchrony index was found $$(F_{1,24} = 430.08, P < 0.001), $$ with an $${\text{R}}^{2}$$ of $$0.78$$ in our full regression model. Moreover, there was a main effect of phase on mean asynchrony $$(F_{1,24} = 269.09, P < 0.001, {\upeta }2{ } = { }0.4), $$ with a 48.82 ms average increase in mean asynchrony during continuation compared to pacing. We also observed an average increase of 8.44 ms in mean asynchrony for syncopation compared to synchronization that was significant $$(F_{1,24} = 5.83, P = 0.02, {\upeta }2{ } = { }0.14)$$, and an average increase of 23.15 ms in mean asynchrony for the alternating compared to block design, which was also significant $$(F_{1,24} = 96.22, P < 0.001, {\upeta }2{ } = { }0.33)$$. Thus, all three independent variables—tapping mode, maintenance phase, and study design—significantly impacted mean asynchrony. Moreover, a significant phase $$\times$$ study design interaction $$(F_{1,24} = 5.97, P < 0.02, {\upeta }2{ } = { }0.24)$$ and mode $$\times $$ study design interaction $$(F_{1,24} = 7.29, P < 0.01, {\upeta }2{ } = { }0.28) $$ were observed.

#### Block study design

**Figure 1:**
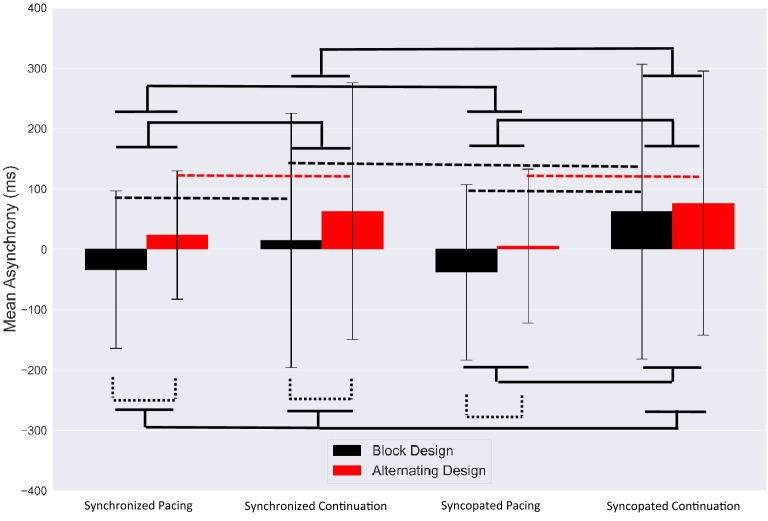
Mean asynchronies of each timing condition for the block design (black) and alternating design (red). Error bars show standard deviation (SD). Dashed brackets indicate statistically significant comparisons between two study designs and solid brackets represent significant contrast effect between timing conditions.

In order to find the accuracy of synchronized and syncopated paced tapping, the average mean asynchronies were measured as $- 33.75 \pm 130.5 \left( {ms} \right)$ (mean $\pm$ SD) and $- 38.03 \pm 145.34{ }\left( {{\text{ms}}} \right)$, respectively (see Fig. 1). This clearly shows negative mean asynchrony (NMA) (i.e., the anticipation of tapping response with respect to the auditory stimulus)^3^ in the pacing phase for synchronization and syncopation. Moreover, the average continuation phase with no metronome present was estimated at $14.71 \pm 210.8{ }\left( {{\text{ms}}} \right)$ for synchronized tapping and $62.91 \pm 244.25{ }\left( {{\text{ms}}} \right){ }$ for syncopated tapping conditions. This indicates more variability for continuation than pacing in both tapping modes.

By using Tukey’s HSD posthoc test, we observed significant differences between pacing and continuation in synchronization mode ($F_{1,24} = 8.18, P < 0.001, {\upeta }2{ } = { }0.24)$; between phases in syncopation $F_{1,24} = 18.62, P < 0.001, {\upeta }2{ } = { }0.34$; and synchronization and syncopation in continuation phase ($F_{1,24} = 8.26, P < 0.001, {\upeta }2{ } = { }0.23)$.

#### Alternating study design

For participants in the alternating study design, for synchronized and syncopated pacing and continuation, the mean asynchronies were $24.03 \pm 106.22{ }\left( {{\text{ms}}} \right)\left( {{\text{mean}} \pm {\text{SD}}} \right),$ $5.33 \pm 127.93{ }\left( {{\text{ms}}} \right)$, $62.99 \pm 212.73{ }\left( {{\text{ms}}} \right)$, and $76.59 \pm 218.92{ }\left( {{\text{ms}}} \right)$, respectively, as illustrated in Fig. 1. The results reveal that pacing resulted in more accurate and stable tapping compared to the continuation phase. Significant differences between pacing and continuation were observed for synchronization $(F_{1,24} = 6.13, P < 0.001, {\upeta }2{ } = { }0.13)$ and syncopation $(F_{1,24} = 12.46, P < 0.001, {\upeta }2{ } = { }0.33){ }$ tapping modes.

Specifically, we found a significant contrast effect between pacing and continuation phases ($\left( {F_{1,24} = 23.65, P = 0.001, {\upeta }2{ } = { }0.2} \right)$, and also between the block and alternating study designs when averaging across the two tapping modes $\left( {F_{1,24} = 14.96, P = 0.001, {\upeta }2{ } = { }0.18} \right)$. Significant contrast effects were observed between the block and alternating design in synchronized pacing $\left( {F_{1,24} = 9.04, P < 0.001, {\upeta }2{ } = { }0.31} \right)$, synchronized continuation $\left( {F_{1,24} = 8.24, P < 0.001, {\upeta }2{ } = { }0.28} \right)$, and syncopated pacing $\left( {F_{1,24} = 8.27, P < 0.001, {\upeta }2{ } = { }0.29} \right)$. We also observed a contrast effect between pacing and continuation in synchronization $\left( {F_{1,24} = 10.8, P = 0.001, {\upeta }2{ } = { }0.34} \right)$ and syncopation $\left( {F_{1,24} = 22.4, P < 0.001, {\upeta }2{ } = { }0.4} \right)$ when controlling for study design. A contrast effect was also observed for synchronization and syncopation in the continuation phase $\left( {F_{1,24} = 7.5, P < 0.001, {\upeta }2{ } = { }0.3} \right)$ when controlling for study design.

**Figure 2:**
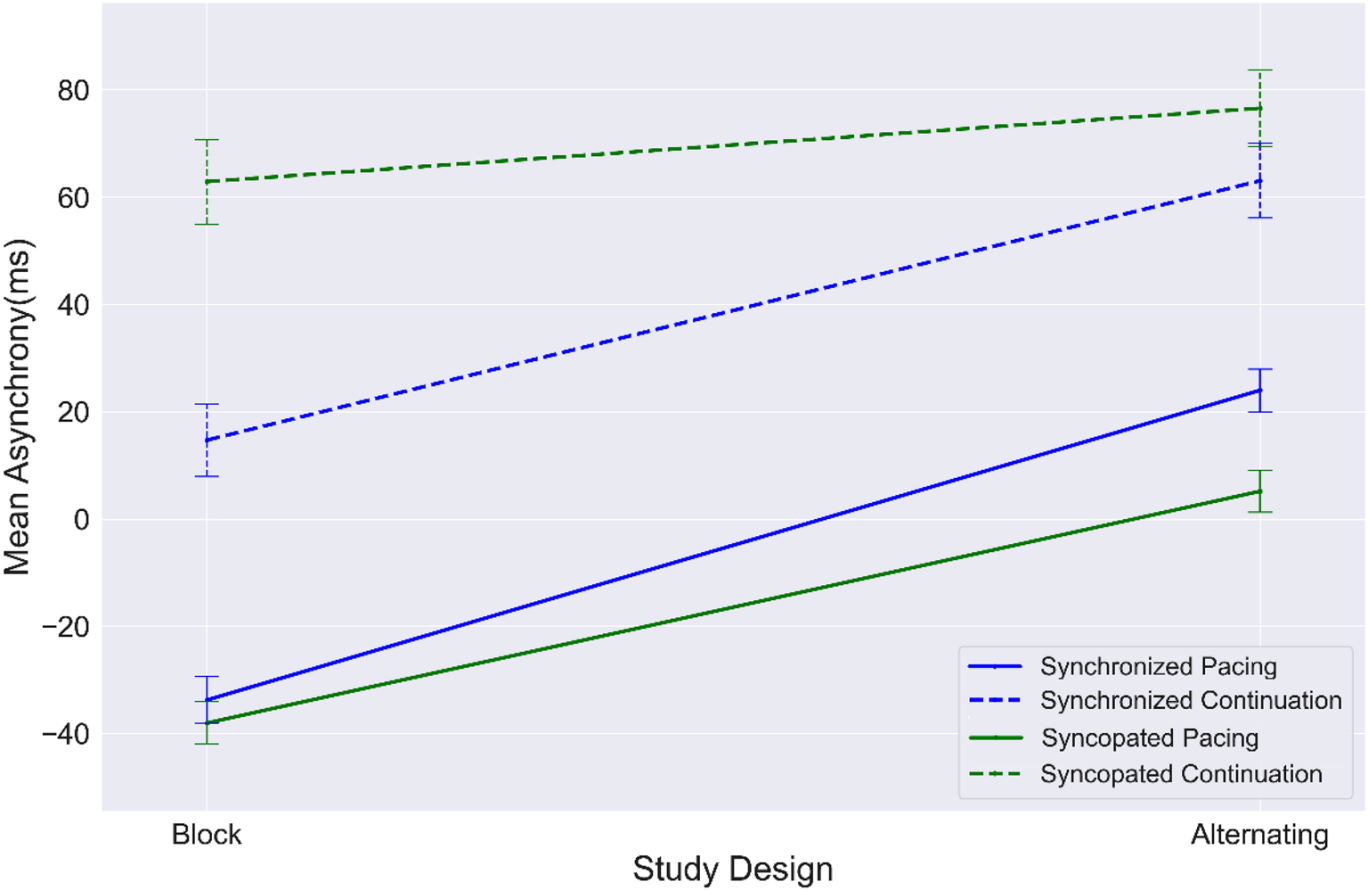
Values of mean asynchrony from block design to alternating design for each timing condition: synchronization (blue); syncopation (green); pacing (solid line); continuation (dashed line). Error bar indicates standard error (SE).

As can be seen in Fig. 2, the average mean asynchrony index in syncopated and synchronized continuation is higher than in pacing for both tapping modes. This is consistent with our previous findings^8^ that more complex timing behavior results in less accurate behavioral performance.

#### Temporal trend and causal inference on mean asynchrony

**Figure 3:**
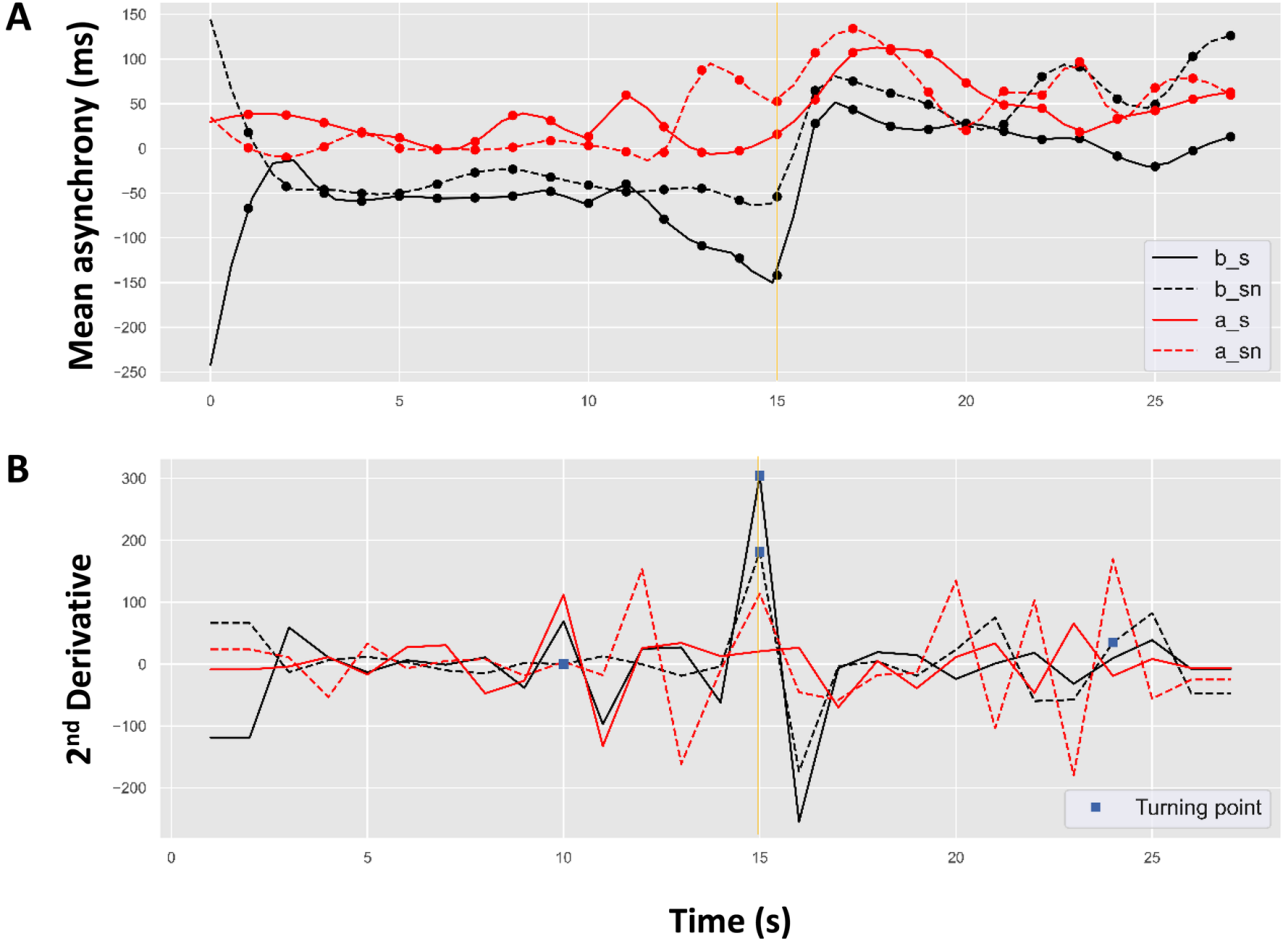
(**A**) Estimated asynchrony trend during maintenance (pacing followed by continuation) and (**B**) second derivatives of the corresponding trends locating the turning points (blue square marks) in: blocked synchronization (b_s as solid black line); blocked syncopation (b_sn as dashed black line); alternating synchronization (a_s as solid red line); and alternating syncopation (a_sn as dashed red line). Gold vertical solid line represents continuation phase onset.

**Figure 4:**
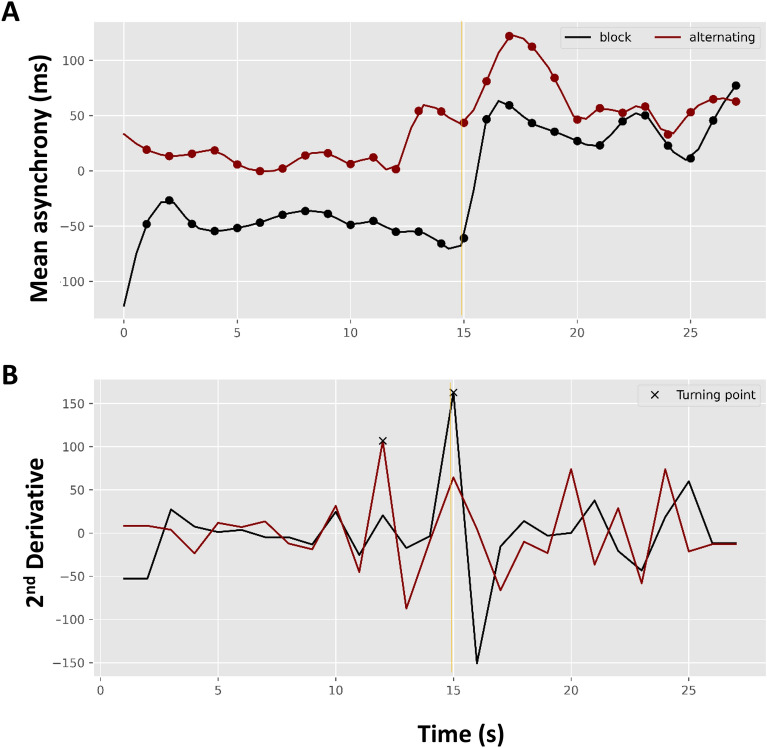
(**A**) Estimated asynchrony trend for the maintenance paradigm (pacing followed by continuation) averaged for tapping mode, and (**B**) second derivatives of the corresponding trends locating the turning points (x-marks) in the block design (black line) and alternating design (red line). The gold vertical solid line represents continuation phase onset.

We used cubic spline interpolation to estimate the fitted model of averaged pacing and continuation tapping trial cycles, as shown in Figs. 3 and 4. Figure 3.A shows the temporal trends for the averaged tapping cycles corresponding to the two tapping modes across the two study designs. The trend for synchronization over time shows a lower level of mean asynchrony than for syncopation; however, an incremental increase in asynchrony in the alternating compared to the block design was observed in all conditions, particularly at the continuation onset time point. Figure 3.B shows the second derivative of the trends specifying the turning point estimated by our interpolated model. As can be seen, the estimated turning point for the block design falls (accurately) at the continuation onset time point (15 s). However, for the alternating study design, the turning point for both synchronized and syncopated tapping occurs at 10 s (5 s before the phase transition time point) and again at 24 s (9 s after phase transition).

Figure 4.A depicts the temporal trends for the averaged tapping cycles corresponding to the two study designs averaged across the tapping modes. The temporal trend for the alternating design has a higher asynchrony level than the block design; however, the incremental growth of asynchrony is observed in both, particularly in the transition to the continuation phase. Figure 4.B shows the second derivative of the estimated asynchrony trends (Fig. 4 A) in which the maximum value represents the turning point of our model (see section Cubic Spline Growth Curve Model). As shown, the estimated turning point for the block design occurs at the continuation onset time point (second: 15). However, the turning point for the alternating design appears at time point 12 (3 s before the phase transition).

**Figure 5:**
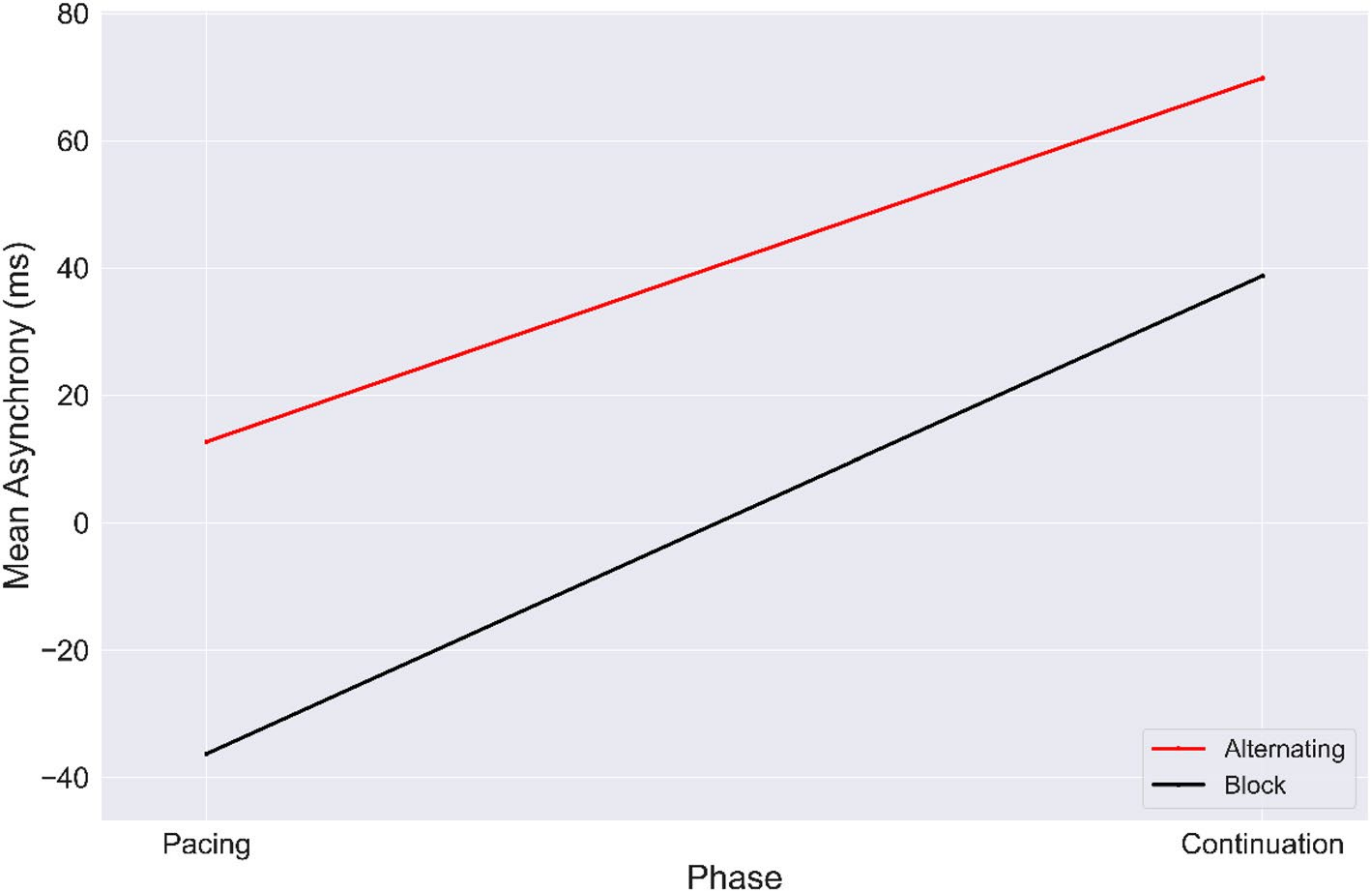
Graphic illustration of the DID estimator. Values of mean asynchrony averaged by tapping modes from pacing to continuation for each study design: block (black) and alternating (red).

As shown in Fig. 5, the DID approach estimated by OLS was implemented to find the causal effect of study design on what we will refer to as rhythmic entrainment (Kurtosis = 3.03, Skewness = -0.1). From a total of 7842 observations (trials), we observed a significant causal effect of study design on mean asynchrony output $$(F_{2,7839} = 187.02, P < 0.001), $$ with an $${\text{R}}^{2}$$ of $$0.04$$. As shown in Eq. (3), four defined regression coefficients were used to estimate the effect of each of the following on the outcome: $$\beta_{0}$$: baseline mean asynchrony during pacing for block design; $$\beta_{1}$$: mean asynchrony temporal changes of block design; $$\beta_{2}$$: mean asynchrony temporal changes of alternating design; $$\beta_{3}$$: interaction of mean asynchrony between the two study designs. The coefficients were estimated as reported in Table 1. As shown, all coefficients were significant. These results demonstrate the causal impact of the study design on phase of the continuation paradigm.

**Table 1:** Basic DID models of mean asynchrony for study design causality.

	$\beta$	SE	T statistics	R
Constant	− 36.12	1.22	− 5.343*	0.3
Block	75.21	3.392	16.213**	0.66
Alternating	54.33	4.107	9.543**	0.33
Block $\times$ alternating	− 10.19	4.113	9.796**	0.63
Obs			7842	
BIC			442.33	
Likelihood-ratio test of alpha = 0			Chibar2(01) = 883.3 Prob $\ge$ chibar2 = 0.000	

*SE* standard error, $\beta$, Regression coefficient**,** Prob Probability, obs Observation, *BIC* Bayesian information criterion.

*$$P < 0.01$$, **$$P < 0.001$$.

#### IRI

MLR was also used to predict IRI based on timing type, maintenance phase, and study design; the outcome was significant $$(F_{1,24} = 366.28, P < 0.001), $$ with an $${\text{R}}^{2}$$ of $$0.52$$ in our full regression model. We observed a main effect of phase on IRI $$(F_{1,24} = 166.05, P < 0.001, {\upeta }2{ } = { }0.42) $$ with superior performance for pacing relative to continuation. We also found an average increase of IRI $$\left( {\Delta IRI = {13}.{\text{77 ms}}} \right)$$ during syncopation relative to synchronization $$(F_{1,24} = 98.05, P < 0.001, {\upeta }2{ } = { }0.44)$$, and an average increase $$\left( {\Delta IRI = {15}.{\text{44 ms}}} \right)$$ for the alternating compared to block design $$( F_{1,24} = 14.16, P < 0.001, {\upeta }2{ } = { }0.22)$$, thus favoring block design for accuracy. Likewise, all three independent variables—phase, tapping mode, and study design—significantly impacted IRI. Moreover, the phase and study design significantly interacted with each other $$(F_{1,24} = 16.3, P < 0.001, {\upeta }2{ } = { }0.14)$$.

#### Block study design

**Figure 6:**
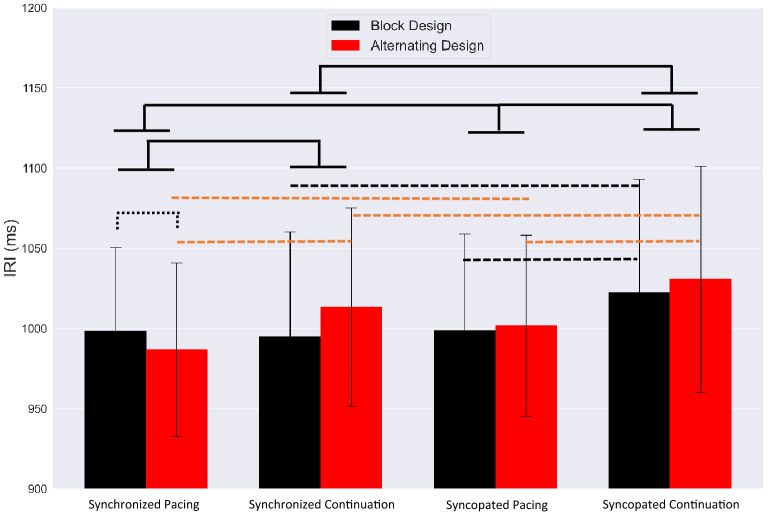
Averaged IRIs with SDs for each condition for block (black) and alternating designs (red). Dashed brackets indicate statistically significant comparisons between the two study designs and solid brackets represent significant contrast effects between timing conditions.

The mean IRIs and SDs are depicted in Fig. 6. Synchronized tapping was performed with a mean IRI of $998.32 \pm 52.24$ (ms) and $994.8 \pm 65.34$ (ms) for the pacing and continuation phases, showing performance was very close to on time metronome. The average response rate was slower during syncopated tapping, with a mean IRIs of $998.76 \pm 59.93{ }$ (ms) for pacing and $1022.18 \pm 70.7{ }$ (ms) for continuation phases.

Post-hoc analyses of our regression model revealed a significant contrast effect for syncopated pacing and syncopated continuation $\left( {F_{1,24} = 10.2, P < 0.01, {\upeta }2{ } = { }0.28} \right)$. We also observed a contrast effect for synchronized continuation and syncopated continuation $\left( {F_{1,24} = 11.23, P < 0.001, {\upeta }2{ } = { }0.43} \right)$.

#### Alternating study design

Synchronized tapping was performed with a mean IRI of $986.65 \pm 54.03$ (ms) and $1013.41 \pm 61.69{ }$ (ms) for the pacing and continuation phases. Moreover, during syncopated tapping, the average response rate was slower, with a mean IRIs of $1001.76 \pm 56.42{ }$ (ms) for pacing and $1030.75 \pm 70.51{ }$ (ms) for continuation phases.

A post-hoc analysis of the linear regression model for the alternating design revealed a contrast effects at every level for the alternating study design: synchronized pacing and synchronized continuation $\left( {F_{1,24} = 11.32, P < 0.001, {\upeta }2{ } = { }0.34} \right){ }$, syncopated pacing and syncopated continuation $\left( {F_{1,24} = 16.18, P < 0.001, {\upeta }2{ } = { }0.37} \right)$, synchronized pacing and syncopated pacing $\left( {F_{1,24} = 5.41, P < 0.001, {\upeta }2{ } = { }0.19} \right)$, synchronized continuation and syncopated continuation conditions $\left( {F_{1,24} = 8.19, P < 0.001, {\upeta }2{ } = { }0.27} \right)$ (see Fig. 6).

We observed a significant contrast effect for synchronization and syncopation tapping modes $\left( {F_{1,24} = 11.15, P = 0.001, {\upeta }2{ } = { }0.28} \right)$, as well as for the block and alternating study designs $\left( {F_{1,24} = 6.02, P = 0.001, {\upeta }2{ } = { }0.22} \right)$. A main effect of tapping mode $\left( {F_{3,36} = 85.01, P < 0.001, {\upeta }2{ } = { }0.4} \right)$ and study design $\left( {F_{1,24} = 14.88, P < 0.001, {\upeta }2{ } = { }0.33} \right)$ on the IRI marker was also observed. We also observed an interaction between tapping mode and study design on IRI. $\left( {F_{3,36} = 15.32, P < 0.001, {\upeta }2{ } = { }0.26} \right)$.

When controlling for tapping mode, we observed a significant difference between the block design and alternating design $\left( {F_{1,24} = 6.02, P = 0.001, {\upeta }2{ } = { }0.22} \right)$. Specifically, a significant contrast effect between block design and alternating design was observed for synchronized pacing $\left( {F_{1,24} = 4.69, P < 0.02, {\upeta }2{ } = { }0.16} \right)$. We also observed a contrast effect between pacing and continuation for synchronization $\left( {F_{1,24} = 6.83, P = 0.001, {\upeta }2{ } = { }0.24} \right){ }$ and syncopation $\left( {F_{1,24} = 18.5, P = 0.001, {\upeta }2{ } = { }0.3} \right)$ tapping modes when controlling for study design. A contrast effect was observed between synchronization and syncopation for the continuation phase $\left( {F_{1,24} = 14.9, P = 0.001, {\upeta }2{ } = { }0.33} \right)$ when controlling for study design.

**Figure 7:**
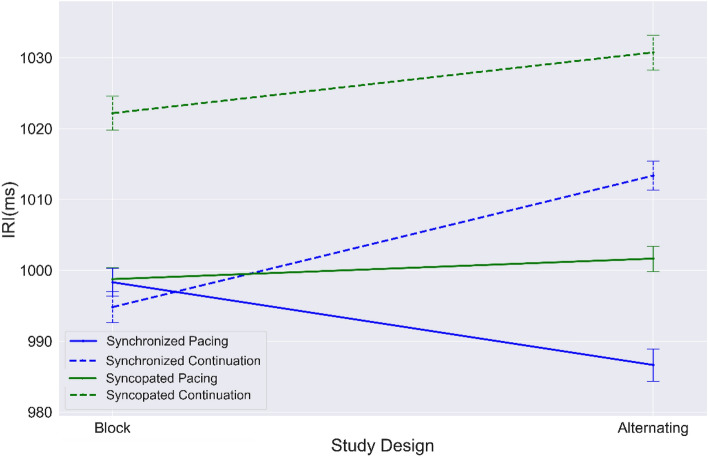
Mean values of IRIs from block and alternating designs for each condition: synchronization (blue); syncopation (green); pacing (solid line); continuation (dashed line). Error bar indicates standard error (SE).

Figure 7 shows that the IRI mean value in syncopated continuation is significantly higher than all other conditions regardless of study design. While supporting our prior findings^8^ that more complex timing behavior leads to less accuracy of behavioral performance, the results also show that participants performed better in the block design than in the alternating design across conditions.

#### Temporal trend and causal inference on IRI

**Figure 8:**
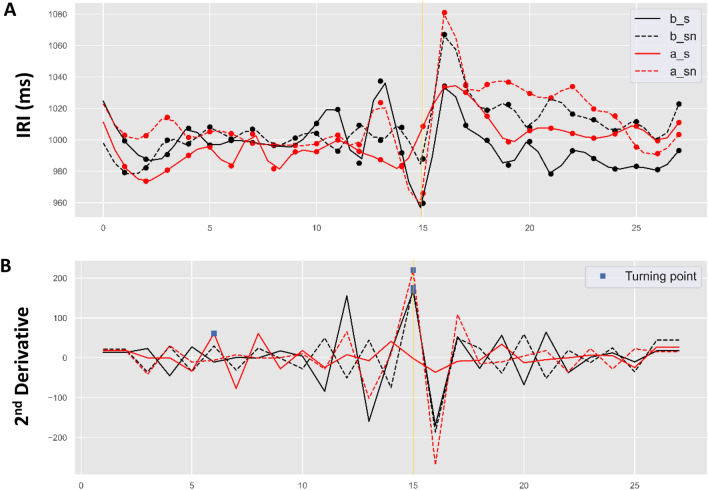
(**A**) Estimated IRI trend of maintenance paradigm (pacing followed by continuation), and (**B**) second derivatives of the corresponding trends locating the turning points (blue square marks) in synchronization + block design (b_s as solid black line); syncopation + block design (b_sn as dashed black line); synchronization + alternating design (a_s as solid red line); and syncopation + alternating design (a_sn as dashed red line). Gold vertical solid line represents continuation phase onset.

**Figure 9:**
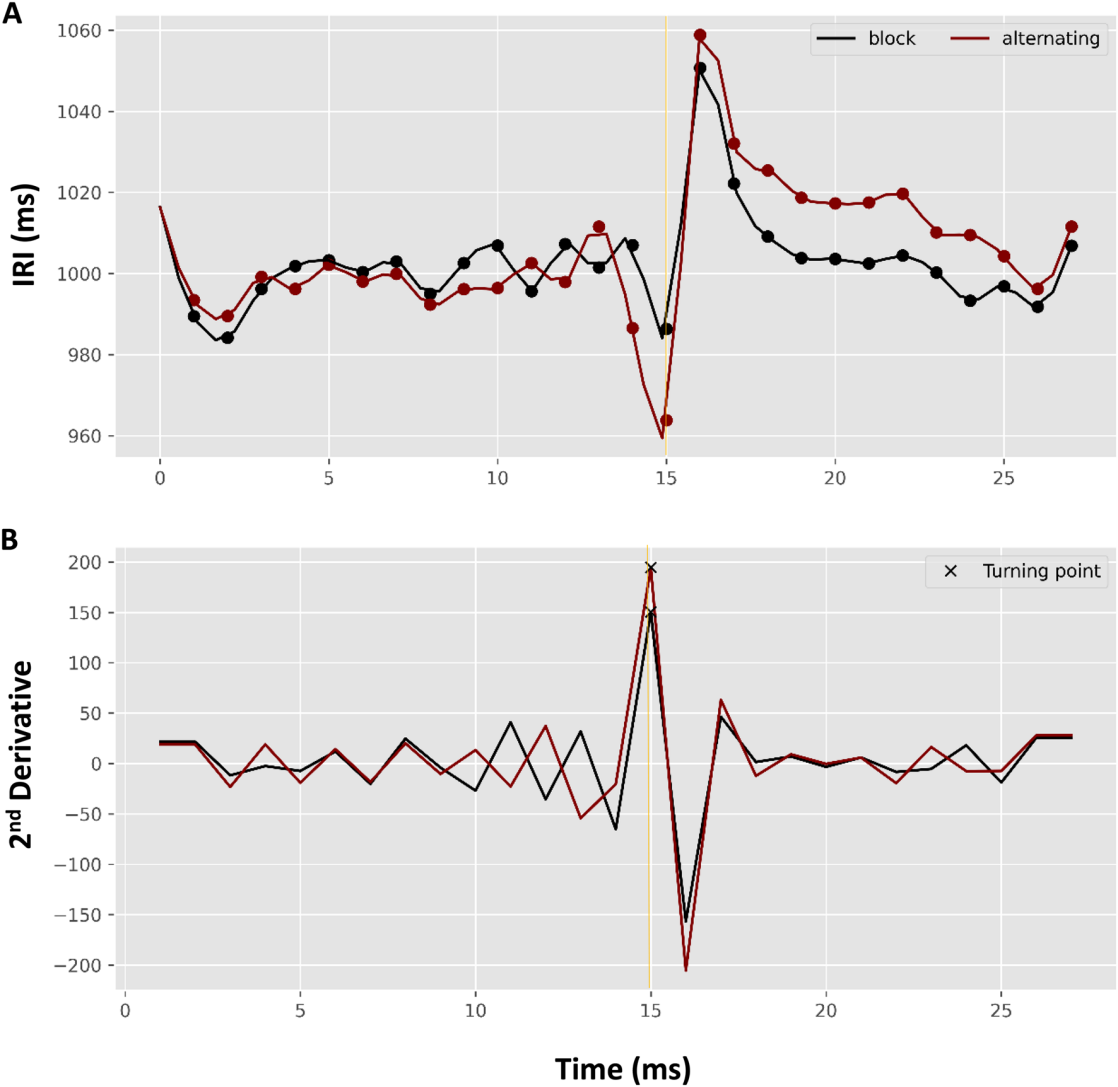
(**A**) Estimated IRI trend for maintenance paradigm (pacing followed by continuation) across tapping mode by design type, and (**B**) second derivatives of the corresponding trends locating the turning points (x-marks) for the block design (black line) and alternating design (red line). Gold vertical solid line represents continuation phase onset.

Using cubic spline interpolation, we estimated the fitted model of averaged pacing and continuation tapping trial cycles, as shown in Figs. 8 and 9. Figure 8 A shows four different IRI temporal trends of the averaged tapping cycles corresponding to the mode and phrase of the two design types. Syncopated tapping in both designs shows an abrupt increase in at 16 s (at first unpaced tap). However, gradual decrease has been observed in all trends during continuation of both study designs. In this regard, as shown in Fig. 8 B, the estimated turning points of all trends occur at the continuation onset time point (15 s); however, the turning point for syncopated tapping occurs at 6 s (9 s before phase transition time point) in the alternating study design.

Figure 9 A depicts temporal traces of the mean IRI cycles corresponding to the two study designs. The temporal trace corresponding to alternating design is higher than the block design in continuation phase; however, the abrupt growth of IRI was observed in both designs in the transition to continuation phase. Figure 9 B shows the second derivative of the trend in which the estimated turning points for both study designs fall on the continuation onset time point (15 s).

**Figure 10:**
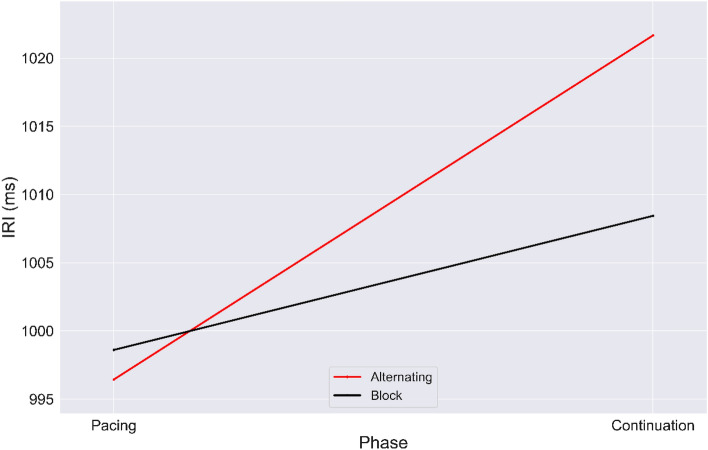
Graphic illustration of the DID estimator. The values of IRIs averaged across tapping mode with pacing to continuation phase for each study design: block (black line); alternating (red line).

We implemented the DID approach estimated by OLS to find the causal effect of study design on accuracy (Fig. 10). From a total 7023 observations, we observed a significant causal effect of the manipulation on mean asynchrony output $$(F_{2,7020} = 73.78, P < 0.001), $$ with an $${\text{R}}^{2}$$ of $$0.02$$ (Kurtosis = 3.170, Skewness = 0.085). Four regression coefficients ($$\beta_{0}$$: baseline mean IRI of block design in pacing period; $$\beta_{1}$$: IRI temporal changes of block study design group; $$\beta_{2}$$: IRI temporal changes of alternating study design; $$\beta_{3}$$: interaction of IRI changes between two study designs) were estimated to determine whether there were the main effects of independent variables (see Causal Inference section for more information). Accordingly, as reported in Table 2, all regression coefficients were significant, revealing the causal effect of study design on the tapping accuracy in the maintenance paradigm.

**Table 2:** Basic DID models of IRI for study design causality.

	$\beta$	SE	T statistics	R
Constant	998.24	0.82	795.011*	0.13
Block	10.29	1.252	10.273**	0.46
Alternating	25.63	1.449	13.255**	0.53
Block $\times$ alternating	15.41	1.502	3.685**	0.33
Obs			7023	
BIC			393.5	
Likelihood-ratio test of alpha = 0			Chibar2(01) = 143.3 Prob $\ge$ chibar2 = 0.001	

*SE* Standard error, $\beta$, Regression coefficient, Prob Probability, obs Observation, *BIC* Bayesian information criterion.

*$$P < 0.01$$, **$$P < 0.001$$.

#### Cortical hemodynamics

Here we investigate the result of the hemodynamic activity for each timing condition in two different methodological designs of the fNIRS experiment (block versus alternating design). We depict and compare group-level channel maps' results via AR-IRLS regression model. All detailed information of regressed HRFs (SM.1), contrast plots (SM.3), and Montreal Neurological Institute (MNI) coordinates corresponding to the channels (SM.4) were reported in the Supplementary Materials. All generated channel maps reported in the Results section and the Supplementary Materials section were generated using the Brain AnalyzIR toolbox.

#### Synchronized pacing

**Figure 11:**
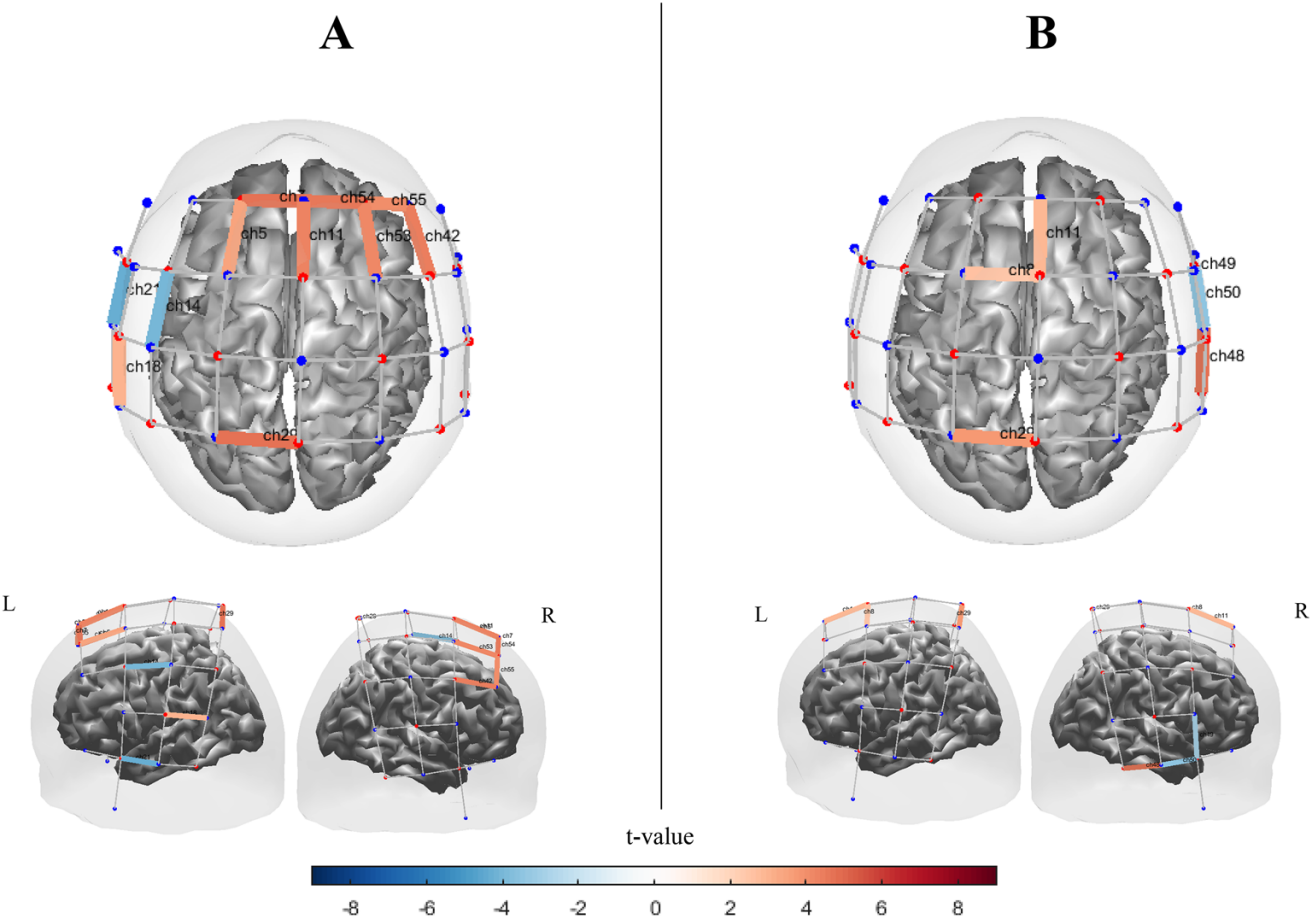
Channel maps of the main effect (q < 0.05) of synchronized pacing condition on [oxy-Hb] hemodynamic activation obtained by (**A**) Block design and (**B**) Alternating design. Color bars represent the t-value range. The channel maps were generated using the Brain AnalyzIR toolbox^54^.

Channels with significant activation for synchronized pacing conditions obtained by block design and alternating design are shown in Fig. 11. In the block design, we observed significant [oxy-Hb] activations in left primary sensorimotor area $\left( {t_{ch29} = 5.2, q = 0 < 0.01} \right)$, and also significant activation in left temporal gyrus $\left( {t_{ch18} = 4.84, q < 0.001} \right)$, right PMC area $\left( {t_{ch11} = 4.64, q < 0.02;t_{ch42} = 4.74, q < 0.02;t_{ch53} = 5.34, q < 0.01} \right)$ extending anteriorly into medial prefrontal area $\left( {t_{ch54} = 5.27, q < 0.001;\;t_{ch55} = 4.2, q = 0.02} \right)$, and SMA $\left( {t_{ch11} = 5.024, q = 0 < 0.01} \right)$ regions in block design (Fig. 11 A). Moreover, significant inverse activations were observed in left primary sensorimotor area $t_{ch14} = - 4.14, q = 0.01$ and $t_{ch21} = - 4.03, q < 0.01$ ; however one significant activation in right temporal area $(eg., t_{ch48} = 6.2, q = 0.01)$ and lower significant activation in motor and frontal areas $(t_{ch8,11} = 3.44, q = 0.04)$ and two inverse activations in right temporal area $(t_{ch19.50} = - 3.9, q = 0.02)$ was observed in alternating design (Fig. 11 B). Significant contrast effects between two study designs were observed mostly in left sensorimotor and right frontotemporal areas (Figure S6).

#### Synchronized continuation

**Figure 12:**
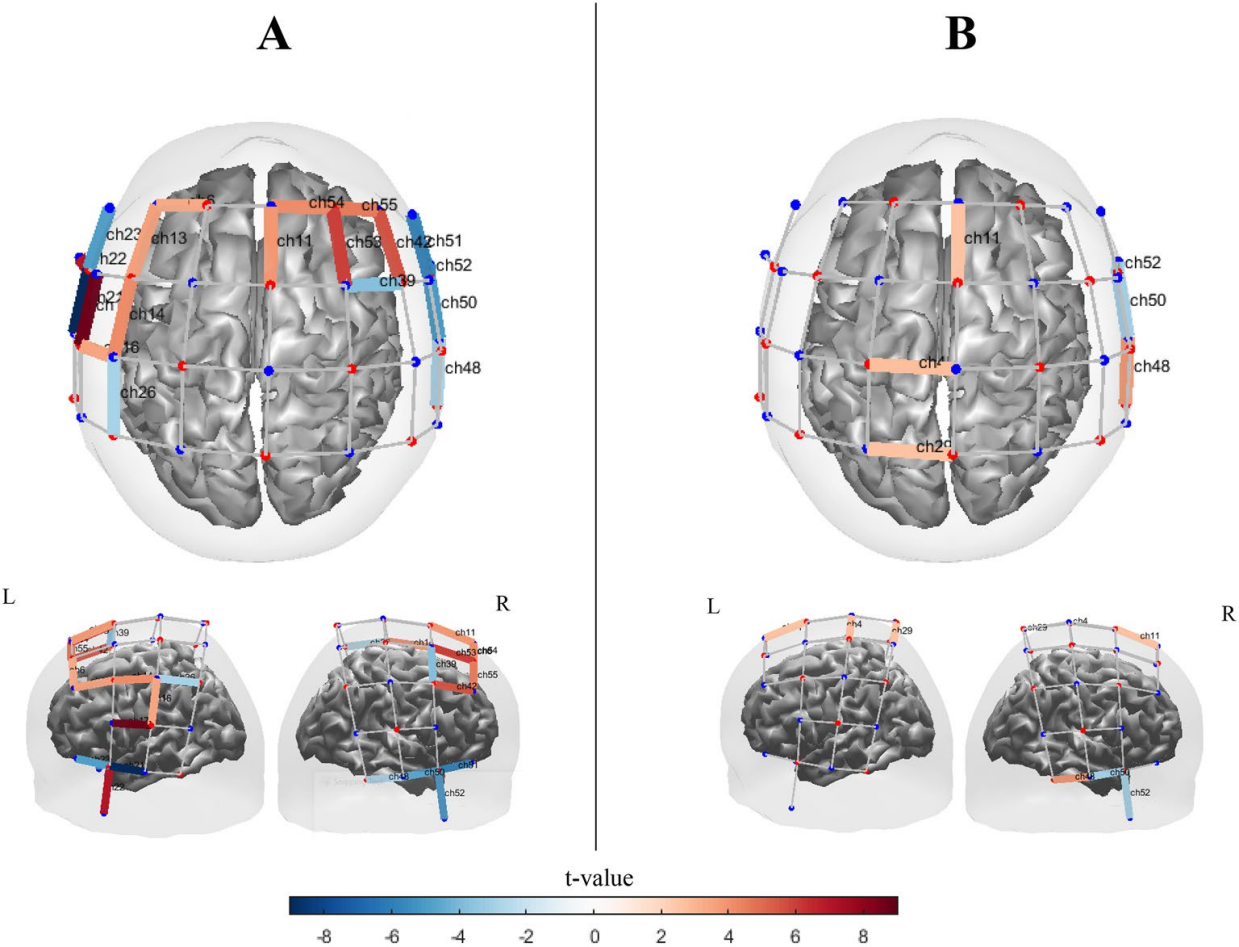
Channel maps of the main effect (q < 0.05) in synchronized continuation condition obtained by (**A**) block design and (**B**) alternating design. Color bars represent the t-value range. The channel maps were generated using the Brain AnalyzIR toolbox^54^.

In Fig. 12, we saw different patterns, with activation of channels in both the block and alternating designs, although there were several significantly activated channel within the left frontal and temporal regions $\left( {t_{ch6} = 3.8, q = 0.04;t_{ch13} = 4.03,q < 0.03;t_{ch14} = 4.03,q < 0.03;t_{ch16} = 3.76,q < 0.04;t_{ch17} = 8.01,q < 0.01;t_{ch22} = 7.8,q < 0.01} \right)$ and [similar to synchronized pacing] right PMC and prefrontal area $\left( {t_{ch54} = 5.3, q < 0.02;t_{ch53} = 6.6;q < 0.01;t_{ch55} = 5.7,q < 0.02; t_{ch42} = 6.7,q < 0.01} \right)$ in the block design. We also observed significant activation in the SMA area $\left( {t_{ch11} = 5.1, q < 0.02;} \right)$. The highly inverse activated area has been observed in left primary temporal areas $(t_{ch21} = - 8.03, q < 0.01;t_{ch23} = - 4.3, q < 0.03;)$ as well as right temporal area $\left( {t_{ch48} = - 3.74, q < 0.05;t_{ch50} = - 5.1, q < 0.03;t_{ch51} = - 6.21, q < 0.03;t_{ch52} = - 6.14, q < 0.03} \right).$ However, the few significant activation has been particularly observed in SMA area $\left( {t_{ch11} = 3.61, q < 0.05} \right)$,left primary motor areas $(t_{ch4} = 3.9, q < 0.05;t_{ch29} = 4, q < 0.05)$ and right temporal area $\left( {t_{ch48} = 5.1, q < 0.04;inverse activations: t_{ch50} = - 4.3, q < 0.04; t_{ch52} = - 4.1, q < 0.05} \right)$ in alternating design. Figure S6 shows significant contrast effects between two study designs which were observed mostly in S1/M1, PFC and temporal areas.

#### Syncopated pacing

**Figure 13:**
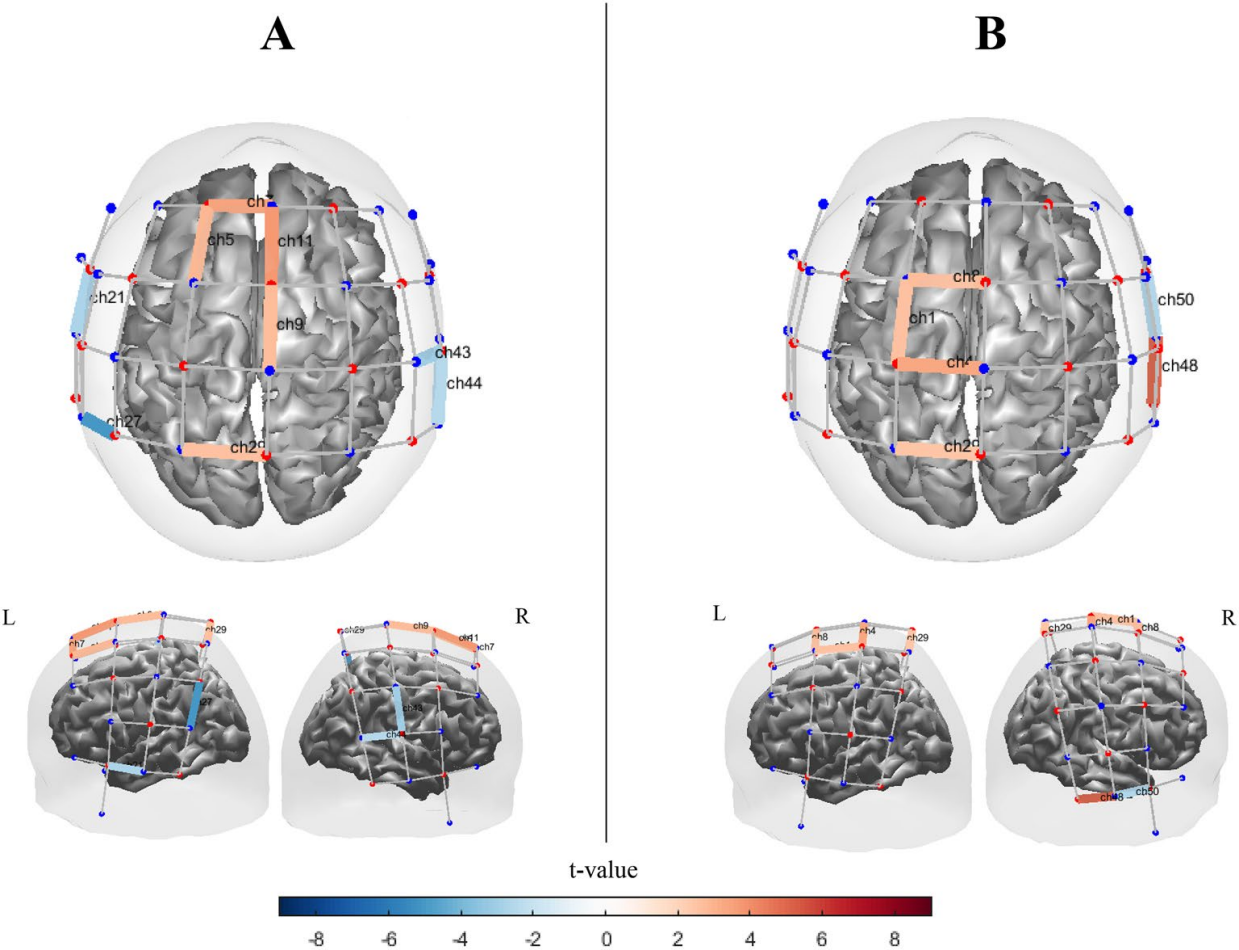
[oxy-Hb] channel maps of the main effect (q < 0.05) in syncopated pacing condition obtained by (**A**) block design and (**B**) alternating design. Color bars represent the t-value range. The channel maps were generated using the Brain AnalyzIR toolbox^54^.

In the block design, the activated channel was observed in SMA $\left( {t_{ch11} = 5.4, q < 0.01} \right)$ and PMC $\left( {t_{ch9} = 4.9, q < 0.05} \right)$. In addition, other significantly activated channels in the block design were observed in the left prefrontal areas $\left( {t_{ch7} = 4.52, q < 0.04;t_{ch5} = 4.3, q < 0.05} \right)$ and left parietal area $\left( {t_{ch10} = 4.04, q = 0.04} \right)$ (Fig. 13 A). We also observed significant inverse activated channel in left and right temporal and parietal areas $\left( {t_{ch21} = - 3.49, q = 0.05;t_{ch27} = - 5.9, q < 0.03; t_{ch43} = - 3.34, q < 0.05;{ }t_{ch44} = 3.51, q < 0.05} \right)$. However, as shown in Fig. 13 B, similar to synchronized pacing condition, few highly significant activated and inverse activated channels for the alternating design are observed. Figure S6 plots significant contrast effects between two study designs which were observed mostly in motor and frontotemporal areas.

#### Syncopated continuation

**Figure 14:**
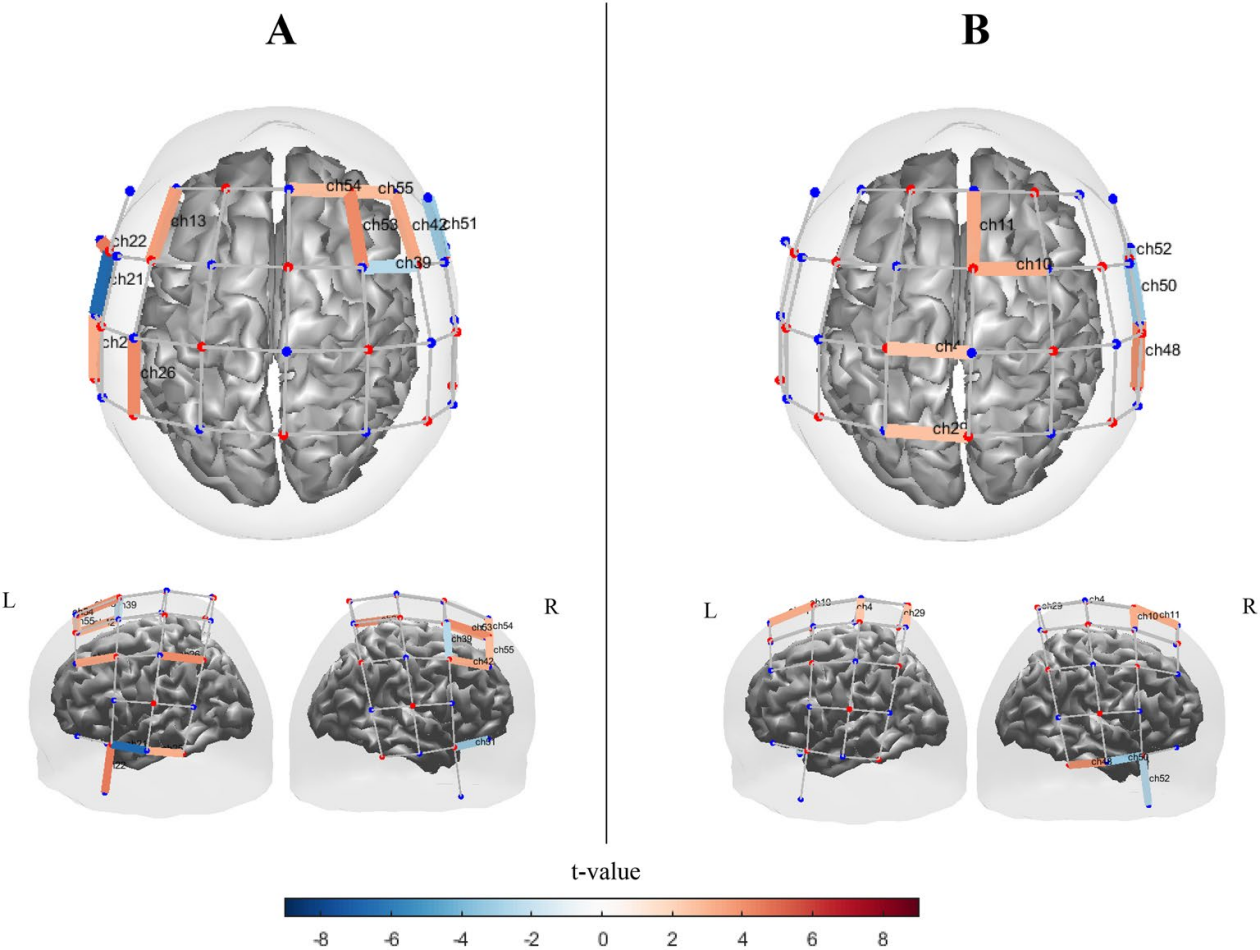
[oxy-Hb] channel maps of the main effect (q < 0.05) in the syncopated continuation condition obtained by (**A**) block design and (**B**) alternating design. Color bars represent the t-value range. The channel maps were generated using the Brain AnalyzIR toolbox^54^.

Syncopated continuation produced several significant channels of activation and inverse activation, particularly in the frontal and temporal areas in the blocked design. However, few highly significant activations and one inverse activation were observed in central and right temporal areas in the alternating design. Figure 14 A illustrates the [oxy-Hb] channel maps with significant main effects in the syncopated continuation condition for the blocked design. As with syncopated pacing, the block design produced stronger [oxy-Hb] activation than the alternating design.

Only one significant area of activation was observed in the right temporal area of the alternating design $\left( {t_{ch48} = 4.53, q < 0.05} \right)$. However, a distribution of channels showing significant levels of activation in central areas ($\left( {t_{ch11} = 4.8, q < 0.04;\;t_{ch10} = 4.6, q < 0.04;t_{ch4} = 3.4, q < 0.05;\;t_{ch29} = 3.7, q < 0.05} \right)$. In contrast, we observed significant activations in left and right frontotemporal areas $\left( {t_{ch13} = 5.2, q < 0.03;t_{ch54} = 4.3, q < 0.05;t_{ch53} = 6.42, q < 0.02;t_{ch55} = 3.6, q < 0.05;t_{ch42} = 4.12, q < 0.04} \right){ }$ and left temporal and parietal areas ($\left( {t_{ch26} = 6: q = 0.02;\;t_{ch25} = 4.6: q = 0.04;\;t_{ch22} = 5.9, q < 0.02} \right)$ for the block design. Moreover, a significantly inverse activated channel in left primary temporal area $\left( {t_{ch21} = - 6.9: q = 0.01} \right)$ and right temporal $\left( {t_{ch51} = - 4.14, q < 0.04} \right)$ and frontotemporal areas $\left( {t_{ch39} = - 4.04, q < 0.05} \right)$. Figure S6 depicts contrast effects between two study designs which were observed widely in sensorimotor, temporal and frontal areas.

## Discussion

The current study aimed to determine whether a subtle manipulation of experimental design would influence participants’ overall accuracy in a tapping task, as well as their corresponding cortical hemodynamics. We measured the behavioral accuracy and hemodynamic activation associated with different timing behaviors. Manipulation of timing behaviors was achieved by manipulating tapping modes (syncopated vs synchronized) and maintenance phase (pacing and continuation) of the task)^7^ across two study designs (blocked vs. alternating). Results showed that both blocked and alternating designs engaged primary temporal and motor areas of the brain, and specific forms of timing behavior differentially engaged frontal and parietal areas. We interpret this as a strong demonstration that study design influenced overall cortical engagement.

We observed significant differences in the behavioral results for both designs between tapping mode and maintenance phase. The causal effect of study design on coordination modes and temporal rhythmic entrainment was demonstrated and established using different timing indices. In our original study, which used a block design^8^, recruitment of a broader cortical network during syncopated continuation compensated for the increased timing demands on the motor system, allowing for stable interval production performance. The differences in neural activity that we observed during blocked synchronized and syncopated tapping may reveal reliance on distinct processing networks in support of more automatic versus cognitively controlled timing behavior. For example, increased activity within subsystems associated with motor planning and preparation (SMA and dorsal-premotor)^10^ and working memory and attention (prefrontal cortex, superior parietal lobe, and MTG)^29^ have been postulated to reflect increases in cognitive demand for performance of the off-beat (syncopated) tapping mode. Such differences were even more pronounced during continuation (when no metronome was present), when the absence of the external auditory stimulus no longer served as a timing guide and cognitive load was greater.

Across both study designs, finger tapping activated cortical areas compatible with the automatic, motor-related timing network (sensorimotor regions). The additional activity observed during syncopated tapping in central, frontal, and parietal areas is consistent with greater engagement of memory and attention processes, and an overall increase in levels of cognitive control. This interpretation is consistent with behavioral findings showing that motor production of anti-phase relationships imposes higher cognitive and attentional demand than in-phase patterns^30,31^. This was observed across both study designs.

A significant difference between the two study designs was observed in the average neural activation as reflected by [oxy-Hb] levels. The stronger neural engagement in central and motor areas was observed in the alternating design context, however it was stronger in both frontal and temporal areas in the blocked design context. These observations were consistent across the timing conditions (SM.3; Figure S6). In particular, the two designs produced different activation patterns for the synchronized versus syncopated modes, and for the pacing and continuation phases. These findings suggest that not only are different cortical networks engaged during externally guided performance of different tapping modes across study designs, but that once established, those different networks continue to operate even when the external guide is no longer present. Although there are likely other mediating factors, one distinct possibility is that these hemodynamic response patterns reflect the differential representation of temporal information given two distinct forms of tapping and the corresponding coordination dynamics. Indeed, process models of interval timing propose specific mechanisms for representing and storing temporal intervals^32^, and the consequence of the existence of two different timing mechanisms is that substantially different networks are recruited to perform the same temporal task^33^. Results presented here strongly suggest that the neural activity supporting the continuation phase not only generalizes across different coordination modes (particularly in complex modes) but that it is also strongly influenced by the study context in which the coordination mode is initially established. Thus, the context of a study’s design plays an integral role in which neural networks are engaged during the pacing-continuation paradigm.

These findings lead to the interesting consequence that the complexity and difficulty of a study’s design (here, blocked vs. alternating) causes different behavioral entrainment and consequently impacts the extent of neural activation, with lower levels of activation observed when entrainment is not allowed to take place, as in the alternating design context. In contrast, greater levels of neural activation were observed in sensorimotor areas across all conditions in the block design. These differences may reflect differences in overall entrainment, with ongoing changes in tapping mode in the alternating study design relative to the block design interrupting any consistent entrainment of motor timing behavior. There is some evidence that hemodynamic responses are modulated by movement parameters^34,35^ further supporting our interpretation that a study’s design impacts context-dependent neural engagement.

The present study interrogates behavioral and brain responses when different rhythmic patterns of motor coordination are introduced into a pacing-continuation task, and further compares responses across a rhythmically blocked versus a rhythmically alternating study design. Behavioral results point to the substantial influence of study design on complex timing behavior. The complexity and difficulty of the particular study design likewise impacts the degree and breadth of neural activation elicited. Significantly higher levels of activity across all four tapping conditions in block design may reflect development of an internal representation of the timing patterns (i.e., entrainment), something that is not possible in the alternating design. Thus, our findings highlight the impact of a study’s design on the degree of rhythmic entrainment that can be achieved given different forms of coordination dynamics. Thus, neural correlates of timing behavior reflect context-dependent parameters.

Our results provide insight into the influence of the broader experimental context on timing behavior and the underlying neural activity that supports it, an interpretation consistent with several previous findings^7,8,36^. Thus, representation of timing information is formed in a context-dependent manner, with the introduction of different cognitive states or expectations, as well as difficulty levels, impacting behavioral performance and the corresponding neural engagement supporting it. Here we have observed that it takes time to develop an internal timing representation and thus entrain motorically to complex rhythmic stimuli.

### Limitations and future work

In our study, we used robust cubic spline growth curve modeling to fit our model to the data and estimate the turning points. However, more timing-based cognitive studies are required to replicate and generalize our findings. Also, detailed mathematical scrutiny can explain the turning point toward and backward displacements across the time in different study design scenarios. Moreover, future research will be needed to understand the details of study design’s impact. For example, an increase in the overall duration of trials in the alternating design context may lead to sufficient entrainment so as to produce comparable levels of hemodynamic activity to those observed in the blocked context. Moreover, contrasting hemodynamic-based measures with other, more direct measures of neurophysiological activity, such as electroencephalography, will be important to further substantiating these findings. Furthermore, there was high inter-subject variability, and therefore low effect sizes in our [oxy-Hb] (One-way ANOVA; $$ F =$$ 21.41, q < 0001,$$\eta^{2} = 0.02$$ ) and [deoxy-Hb] ($$F =$$ 10.49, q < 0001 $$,\eta^{2} = 0.03$$) across all individuals. Specifically in the results obtained from alternating study, we observed significant variability between individuals (for [oxy-Hb]: $$F =$$ 32.29, q < 0001,$$ \eta^{2} = 0.01$$; and [deoxy-Hb]: $$F =$$ 16.43, q < 0001, $$\eta^{2} = 0.01$$); however more stability (low variability) across participants were observed in the results obtained from block design (for [oxy-Hb]: $$F =$$ 8.89, q < 001, $$\eta^{2} = 0.03$$; and [deoxy-Hb]: $$F =$$ 10.69, q < 005, $$\eta^{2} = 0.02$$); Consequently, increasing sample size and using within subject study design (instead of current mixed effect study design would lead to increase power of analysis and decrease inter-subject variability (See section SM.2 in supplementary material for more information).

In addition, we cannot generalize our findings to other cognitive neuroscience studies because of inherent temporal characteristics of action-based timing study which is not affected to most of the neuroscience-based research. Thus, it might be an open question for future research.

## Methods

### Participants

Twenty-three healthy adult volunteers (16 females, 7 males; mean age 26.1, range 19–41) from the University of California, Merced were recruited in the study. Thirteen participants were randomly assigned to perform the tapping task in an alternating design and ten participants were assigned to perform the task in a block design. All were nonmusicians; however, they had finger tapping training before the experiment. Two study design groups were balanced (nonsignificant difference between ages; $F_{1,24} = 33.5, P = 0.3$) and sex (block design: 8 females and 2 males; alternating design: 8 females and 5 males; $F_{1,24} = 0.65, P = 0.1$). All successfully completed the tapping task and participated in fNIRS data collection. All participants were strongly right-handed, according to self-report. No participants reported any neurological or skeletomuscular disorder or injury that would prevent them from performing a timing-based tapping task. Informed consent was obtained from each participant before any data collection.

### Ethics approval

This study was approved by the University of California, Merced Institutional Review Board for research ethics and human participants. Informed consent was obtained from all individual participants included in the study. In addition, all methods were performed in accordance with the relevant guidelines and regulations.

### Task procedure

**Figure 15:**
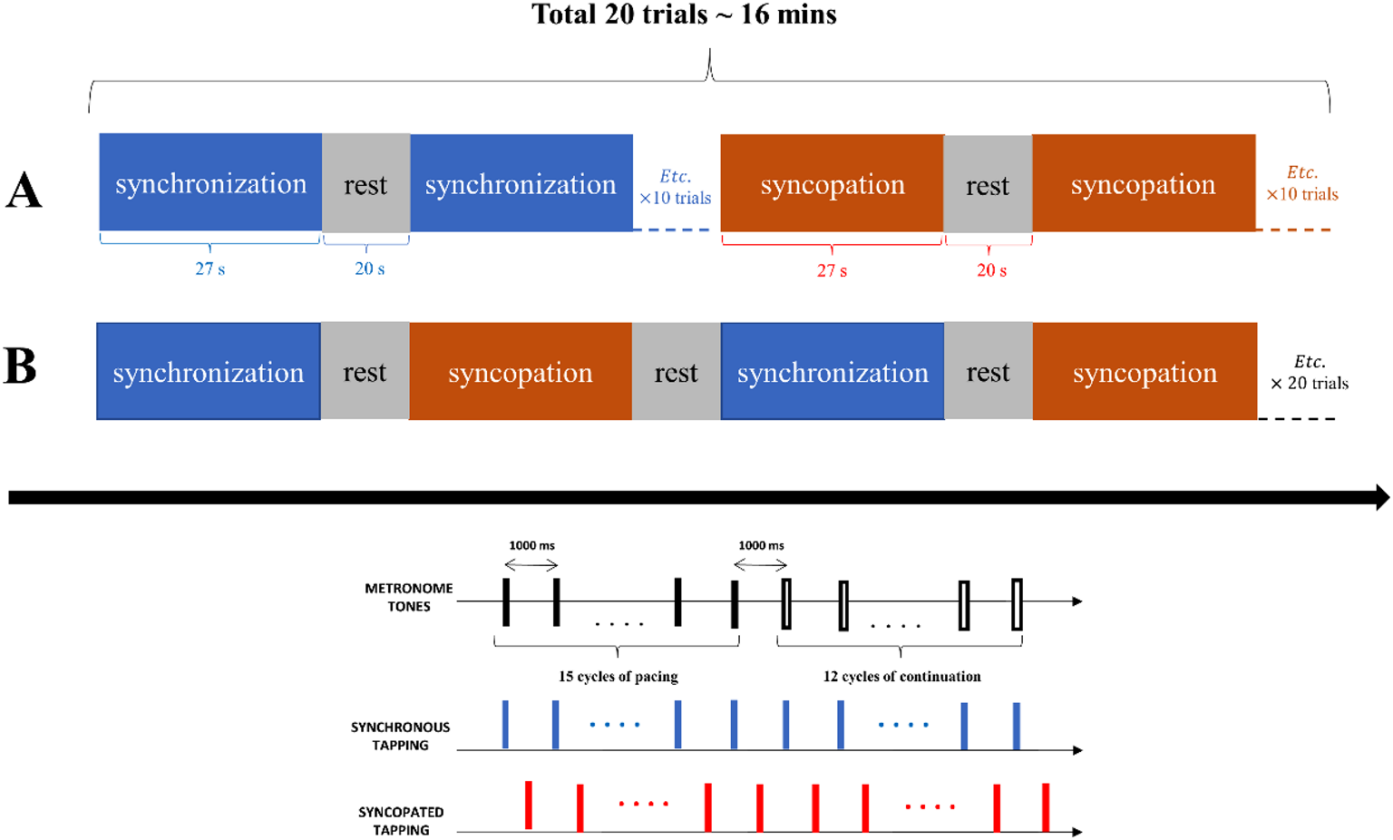
Schematic diagrams stimulus design (**A**) blocked and (**B**) alternating study designs. Participants perform repetitive finger tapping in the presence and then absence of an auditory metronome tone. The task consisted of 20 tapping trials. Each trial began with a pacing phase (15 cycles of tapping with the tone) followed by a continuation phase (12 tapping cycles continued in the same manner established during pacing but without the tone). Tapping patterns were performed in two different coordination modes: synchronized tapping (blue color) or syncopated tapping (red). Ten trials of tapping in each mode were performed corresponding to block or alternating study design. During each trial, participants fixated a crosshair ( +).

Participants performed a rhythmic coordination task in a mixed-method study design containing two different study designs: block design and alternating design (Fig. 15). This task involved tapping on a custom-built metal plate connected to a MakeyMakey™ kit^25,26^ in time with an auditory metronome presented at 1 Hz in order to register the timing of each tap relative to an auditory metronome tone^8^. The time point of a participant's plate finger-tapping was corrected by 25 ms (to correct for temporal device delay due to the time it took the internal circuitry of the MakeyMakey™ to process the input, a built-in delay for input registration that reduces accidental double inputs) to define the onset of each behavioral response.

Two different timing relations were examined: synchronized tapping (pressing on each beat) and syncopated tapping (pressing between successive beats). The order of tapping modes was counterbalanced across the trials (i.e., half of the participants were randomly selected to start with synchronization and the rest with syncopation), and half of the participants completed the tapping task in either blocked (Fig. 15 A) or alternating design (Fig. 15 B). Each tapping mode consisted of two phases of pacing and continuation at a rate of 1 Hz. For each mode, participants were instructed to perform the task condition’s timing relation as best as possible when the metronome was on, and to continue after the metronome stopped. Seated participants performed repetitive right finger movements in the presence of an auditory metronome that produced a 1 kHz tone for 20 ms every 1000 ms (1 Hz). There were ten trials in each condition, with each trial involving 27 cycles (per cycle: 1 s length) of responses (15 cycles of pacing phase and 12 cycles of continuation phase). The time duration of resting states between the trials is 20 s.

## Statistical analysis

### Behavioral analysis

For behavioral analysis (using Python software; libraries: SciPy, Scikit-learn, and Statsmodels), two relative measures of performance were calculated: asynchrony as a measure of accuracy, and inter-response interval as a measure of tapping fluency. We defined asynchrony as the time difference between a participant’s response and the stimulus for each tapping cycle $\left( {tap_{i} - beat_{i} , i \smallint n;cycle number} \right)$^37,38^. We defined “virtual” asynchrony in syncopation as the time difference between a participant’s response and the middle time point of each cycle $\left( {tap_{{mid \left\{ {i,i + 1} \right\}}} - tap_{i} , i \epsilon n;cycle number} \right)$. The term “mean asynchrony” (i.e., coordination error) is used to refer to accuracy for both synchronization and syncopation. Lower mean asynchrony (i.e., an asynchrony value close to 0) indicates higher levels of tapping accuracy. Further, negative mean asynchrony indicates that a participant is predicting the onset of each successive stimulus, while positive mean asynchrony indicates that a participant is reacting to a stimulus onset^3^. Note that virtual mean asynchrony is also defined and computed by considering the taps and the extrapolated silent beats in continuation phases^39^. Lastly, we measured the fluency of tapping, the inter-response interval (IRI), which we defined as the time between consecutive taps $\left( {IRI_{i} = tap_{i + 1} - tap_{i} , i \epsilon n;cycle number} \right)$.

We first removed the outliers of mean asynchrony and IRI indices using the interquartile range (IQR) based method. IQR is defined as the difference between the 25th percentile (Q1) and the 75th percentile (Q3) of our two behavioral markers. We considered an observation to be an outlier if it had a value 1.5 times greater than the IQR or 1.5 times less than the IQR^40^. The average amount of missing data (due to single tap outliers or missing taps) within each timing condition was 3.2 (outliers = 2.98; missing taps = 1.22). We then balanced data using a bootstrapping resampling method by selecting the constant sample size (= 5) within each condition block^41^.

## Regression

We analyzed our data using a multiple linear regression (MLR) model estimated by ordinary least squares (OLS) in order to assess the strength of the association between a set of independent variables (predictors) and single dependent (predicted) variable. Our model defined IRI and mean asynchrony as two separate dependent variables, and phase (pacing vs continuation), tapping mode (synchronization vs syncopation), and study design (blocked vs alternating) as three independent variables. The main equations can be presented as:

1 $$ Y_{Asynchrony} = X.\beta + \varepsilon $$

and

2 $$ Y_{IRI} = X.\beta + \varepsilon $$

where $Y$ is a $n \times 1 \left( {n = 23} \right)$ matrix containing $n$ total observations of each mean asynchrony and IRI variables, the matrix $X$ has dimensions $n \times \left( {k + 1} \right)$ for $three$ independent variables. The vector $\beta_{k} \left( {dimention: \left( {k + 1} \right) \times 1; k\epsilon 0, \ldots ,3} \right)$ and $\epsilon \left( {dimention: n \times 1} \right)$ represent regression coefficient and error, respectively. After observing no violation of MLR assumptions, we estimated our model to predict our mean asynchrony and IRI variables based on our three independent variables and generate the main effect and interaction effect of each independent variable (predictor) on the dependent (predicted) variable.

After estimating our regression coefficients, we used Tukey’s honestly significant difference (HSD) post-hoc test as a multiple pairwise comparison technique^42^ to find the ANOVA contrast effects between all possible group pairing sub categorical observations.

## Cubic spline growth curve model

A flexible statistical approach to model the nonlinear form of temporal trend growth is the piecewise growth curve model (PGCM)^43^. We estimate PGCM by cubic spline interpolation approach^44^ to perform piecewise interpolation and find the turning point of the curve^45^. This allows us to formulate the function of growth forms for pacing and the subsequent continuation phases such that the second derivative of the model represents the turning point of the fitted model. This point is essential since we are interested in comparing growth rates across the pacing and continuation phases. The specification of a turning point is important^45^, since it may happen before or after the intervention (the transition from pacing to continuation).

## Causal inference

Finally, we implemented the difference in differences (DID) method^46,47^ to compare the changes in our behavioral indices over time between averaged indices of the alternating design (as the manipulated group) and the block design (as the control group). We were then able to investigate the causal effect of study design on rhythmic entrainment. The DID technique is ideal for use with the continuation paradigm, given that randomization of pacing and continuation phases at the level of the individual is impossible. No violation of assumptions has been observed in our DID approach. First, the functions and covariates in the model are correctly defined. Second, the expectation value of the error term is zero and the corresponding distributions of covariates are independent. Third, both study designs follow the same trends over time in the absence of intervention. The DID regression model was implemented as shown in the following equations:

3 $$ Y = \beta_{0} + \beta_{1} . \left[ {Block} \right] + \beta_{2} . \left[ {Alternating} \right] + \beta_{3} .\left[ {Block \times Alternating} \right] + \varepsilon $$

‘*Block’* and ‘*Alternating*’ represent control and manipulated groups, respectively, in which dummy variables of time are defined (pacing:0, and continuation:1). The variable ‘$Block \times Alternating$’ is defined as the interaction between behavioral performance changes of block and alternating designs. Also, $\beta_{i} \left( {j\epsilon \left\{ {0,1,2,3} \right\}} \right)$ are defined as regression coefficients of the model. Because tapping mode is randomized over time, we were able to control for its effect and exclude it from the model as a confounding variable.

A post hoc power analysis was conducted using the software package, GPower^48^. The sample size of 23 was used for the statistical power analyses, and a three-predictor variable equation was used as a baseline. The recommended effect sizes used for this assessment were as follows: small $\left( {Cohen^{\prime}s f{ } = 0.14} \right)$, medium $\left( {Cohen^{\prime}s f{ } = { }0.39} \right)$, and large $\left( {Cohen^{\prime}s f{ } = { }0.59} \right)$ (see^49^). The $\alpha -$ level used for this analysis was *p* < 0.05. The post hoc analyses revealed the statistical power for this study was 0.1 and 0.8 for detecting a small and medium effects, respectively, whereas the power exceeded 0.82 for detecting a moderate to large effect size. Thus, there was sufficient power (i.e., power = 0.8) at the moderate to large effect size level, although less than adequate statistical power at the small effect size level.

## fNIRS instrument and analysis

**Figure 16:**
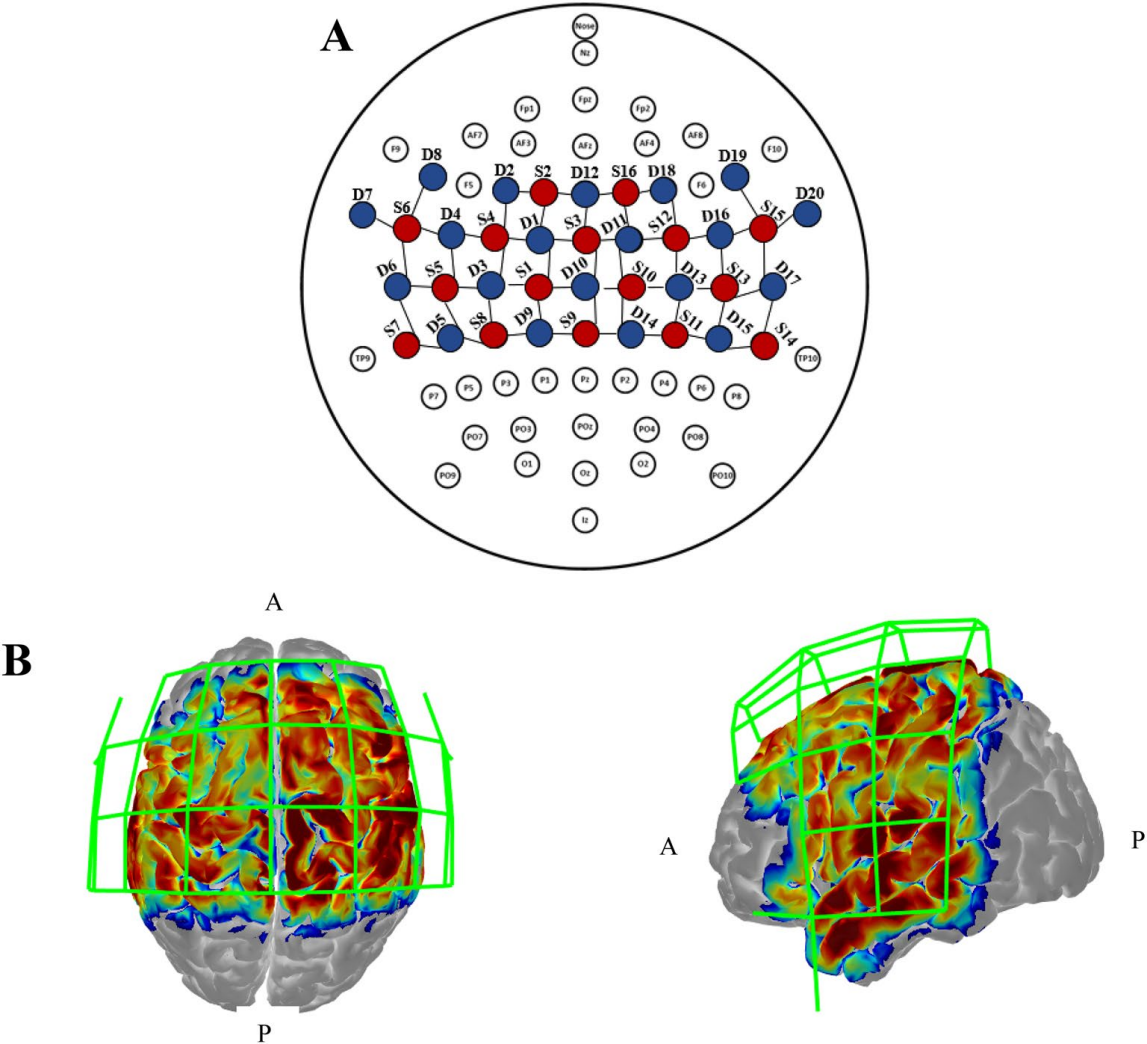
fNIRS probes and channel placement adapted from^8^. Depiction of the geometrical layout of sources (S, red) and detectors (D, blue) concerning the international 10–10 EEG system (**A**) and the corresponding sensitivity maps (**B**) of the probe in a 3D head model. A and P indicate anterior and posterior, respectively. The sensitivity map was generated by AtlasViewer software^50^.

During the finger-tapping task, cortical hemodynamics were monitored and recorded using a multichannel continuous-wave fNIRS system (NIRScout, NIRx Medical Technologies, LLC) with a probe comprising 16 light-source emitter positions containing 760 and 850 nm LED light and 20 APD light detectors. Data were collected at 3.785 Hz, and the average inter-optode distance was 3 cm^8^. Figure 16 illustrates our fNIRS probes and channel placement mapped relative to typical 10–10 scalp landmarks. Moreover, Table S1 in supplementary material (Section SM.4) showed the MNI coordinates according to the corresponding channel numbers and source-detector pairs estimated by using AtlasViewer^50^.

Channel quality was first assessed using QT-NIRS toolbox (SCI threshold = 0.6, Q threshold = 0.7 and PSP threshold = 0.1; https://github.com/lpollonini/qt-nirs; see^51^). Then PCA-based baseline correction and global signal regression (GSR) were applied to raw intensity signals in order to remove DC shifts and global signal. We also corrected motion artifacts using wavelet filtering (SD threshold: 5, basis function: sym8) to remove outliers (motion) and extra low frequency characteristics. Hereafter, raw data were converted to optical density changes and then oxygenated [oxy-Hb] and deoxygenated [deoxy-Hb] hemoglobin concentration changes via the modified Beer-Lambert law (partial pathlength factor: 6.0)^52^. Both preprocessing and activation analyses were measured using the NIRS Brain AnalyzIR toolbox, a MATLAB-based open-source analysis package^53,54^.

Other extracerebral components such as scalp hemodynamics, heartbeat, and respiration, and remaining motion artifacts were corrected using AR-IRLS thoroughly described in [56; see SM.1 section in supplementary materials for more information] and implemented in the NIRS Brain AnalyzIR software described in^57^. Therefore, our approach makes the algorithm robust to physiological and motion artifacts^58,59^. Channel-wise hemodynamic activities were computed using an autoregressive iterative reweighted least square (AR-IRLS) algorithm^55^, where a canonical model was used to estimate the general linear model's regression coefficients and finally generate the hemodynamic response functions (HRFs).

At the group level, we performed a linear mixed-effects model that included the $\beta$ value (together with t-value) per channel and condition as the dependent variable and independent variable, respectively, to model group-level correlations^60^. For each channel, estimates of the t-value for [oxy-Hb] and [deoxy-Hb] were computed across all trials for all participants. Our analyses only include the significantly active and inverse active channels (i.e., statistically non-zero β) with FDR corrected p-value (q-value). However, we focus our analyses on [oxy-Hb] hemoglobin concentration due to its higher signal-to-noise ratio (SNR) and lower inter-participant variability relative to [deoxy-Hb] (see^8^; also refer to section SM.1 in Supplementary Materials for more details about HRFs of [oxy-Hb] and [deoxy-Hb] and the channel maps).

## Supplementary Information

Supplementary Information.

## Data Availability

The data and code that support the findings of this study are available from the first author, AR, upon request (email: rahimpur@stanford.edu).
