# Supplementary material for: Multiple levels of contextual influence on action-based timing behavior and cortical activation
Source: Sci Rep. 2023 May 2;13:7154. doi: 10.1038/s41598-023-33780-1 (PMC10154340; doi:10.1038/s41598-023-33780-1)
Supplement: Supplementary file 1 — Supplementary Information. [file 41598_2023_33780_MOESM1_ESM.docx]

# **SM.1: Hemodynamic response functions**

1. ***Synchronized Pacing***

**A**


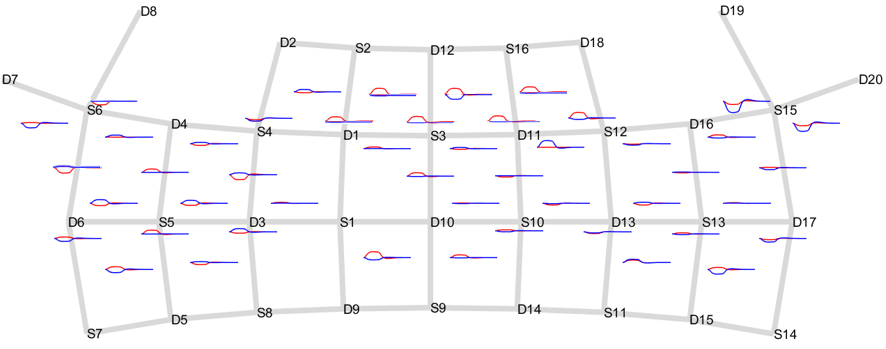


**B**


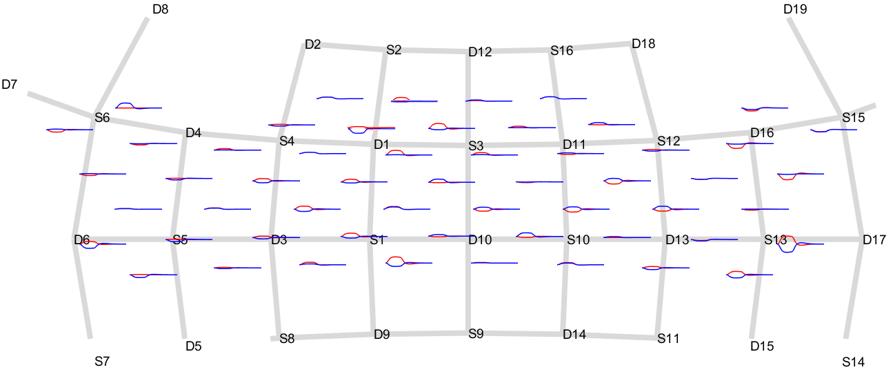


Figure S1. Regression-based hemodynamic response functions of synchronized pacing condition obtained by (A) Block design and (B) Alternating design.

1. ***Synchronized Continuation***

**A**


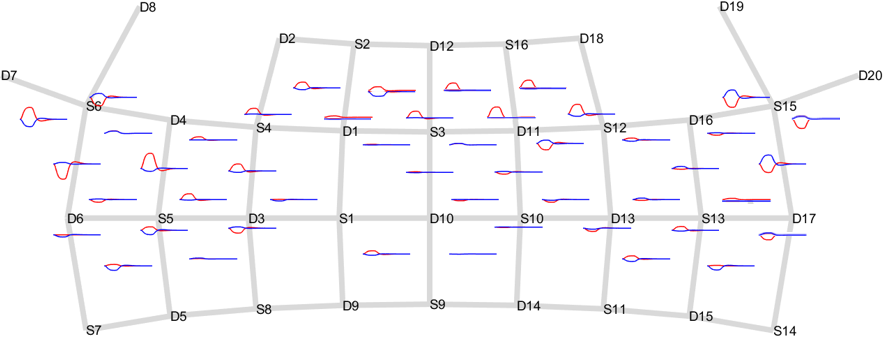


**B**


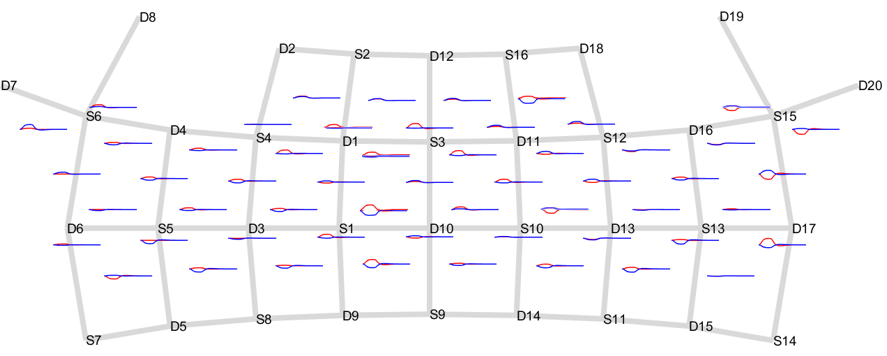


Figure S2. Regression-based hemodynamic response functions of synchronized continuation condition obtained by (A) Block design and (B) Alternating design.

1. ***Syncopated Pacing***

**A**


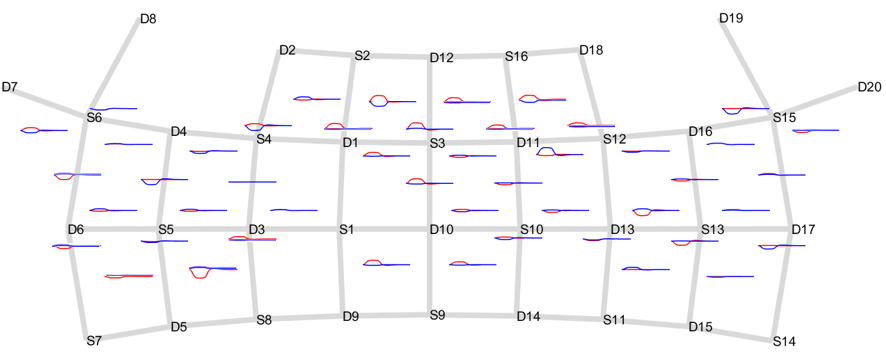


**B**


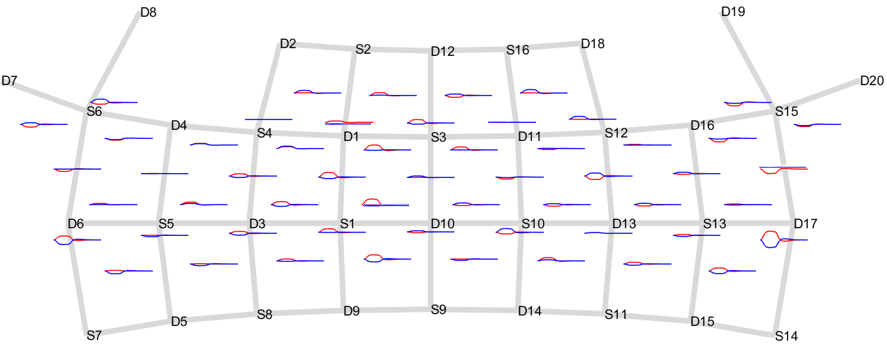


Figure S3. Regression-based hemodynamic response functions of syncopated pacing condition obtained by (A) Block design and (B) Alternating design.

1. ***Syncopated Continuation***

**A**


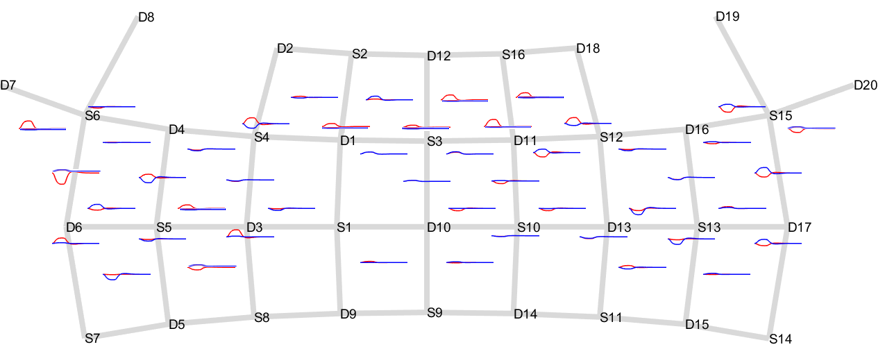


**B**


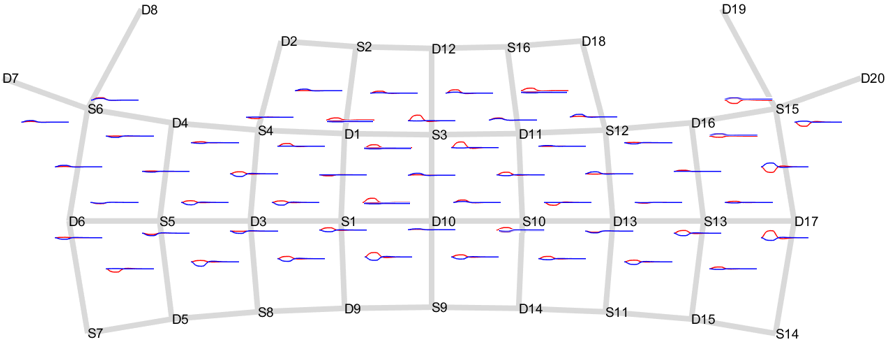


Figure S4. Regression-based hemodynamic response functions of syncopated continuation condition obtained by (A) Block design and (B) Alternating design.

# **SM.2: Inter-subject Variability**


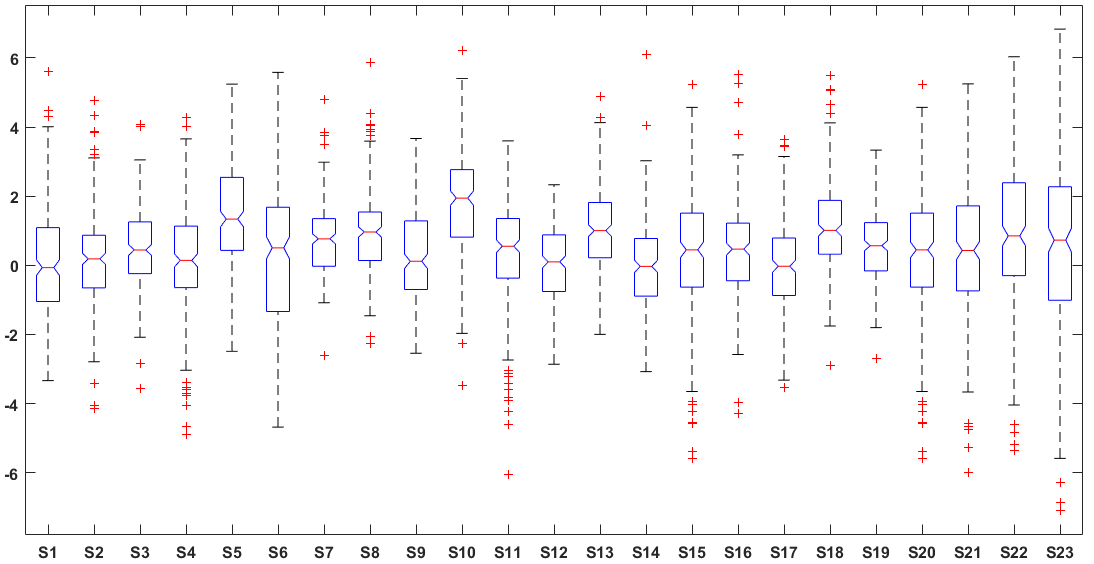


**oxy-Hb Concentration (**$\boldsymbol{\mu Mol)}$


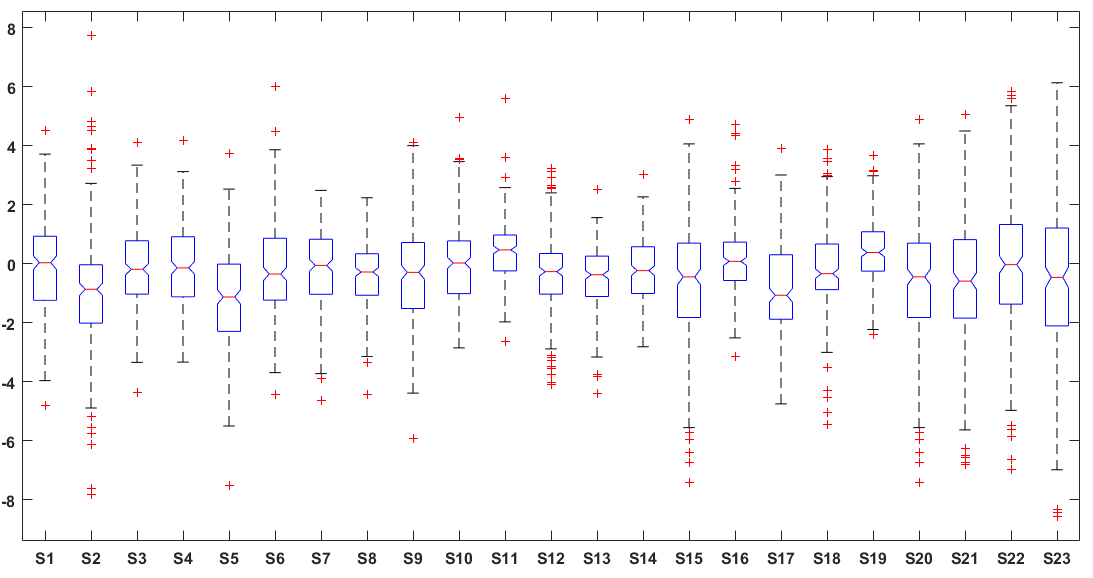


**deoxy-Hb Concentration (**$\boldsymbol{\mu Mol)}$

Performing block design

Performing alternating design

Figure S5. Individual-based bar plots of oxy-Hb (top) and deoxy-HB (bottom) across all timing conditions for 23 subjects (S)

# **SM.3: Contrast Plots**

## ***Alternating design> block design***

| **Synchronized Pacing** | **Synchronized Continuation** |  | |
| --- | --- | --- | --- |
| 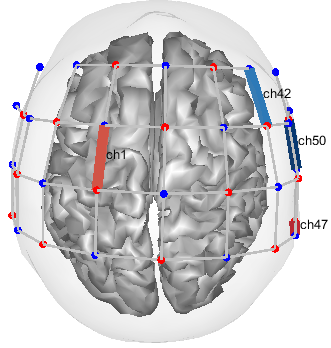  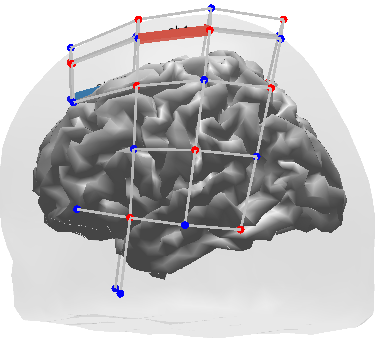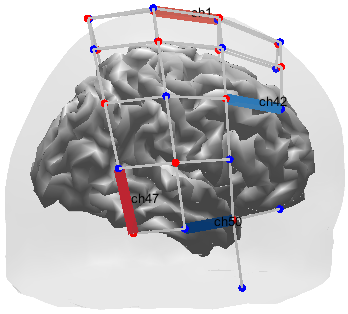 | 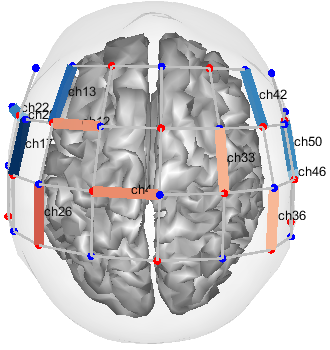  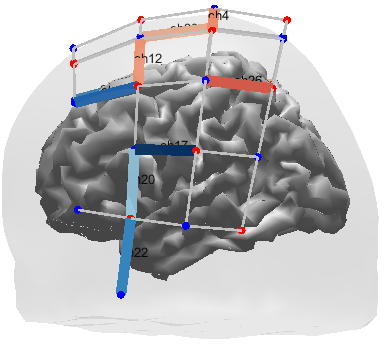 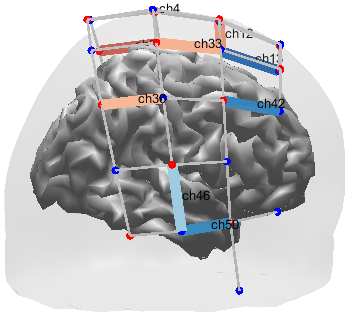 | 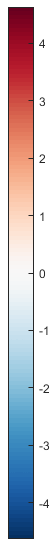 | |
| **Syncopated Pacing** | **Syncopated Continuation** | |  |
| 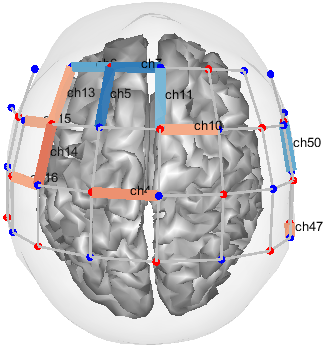  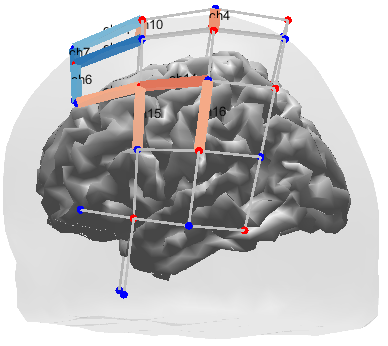 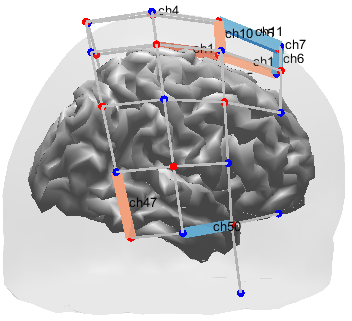 | 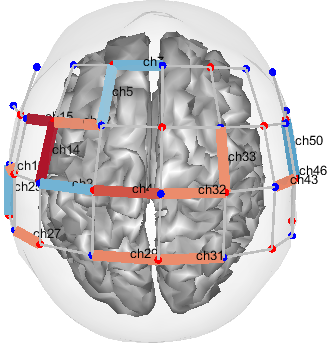  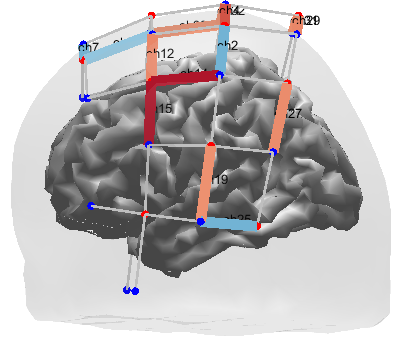 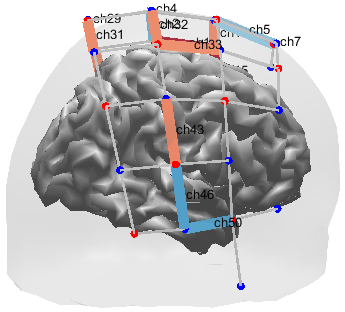 | | 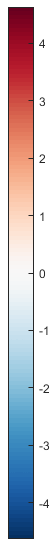 |

Figure S6. [oxy-Hb] channel maps of the contrast effect (alternating> block design; q<0.05) in each condition. Color bars represent the t-value range. The channel maps were generated using the Brain AnalyzIR toolbox.

## ***Contrast maps of timing conditions in block design***

| **Synchronized Continuation > Synchronized Pacing** | **Syncopated Continuation > Syncopated Pacing** |  |
| --- | --- | --- |
| 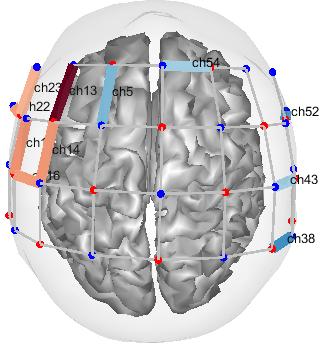  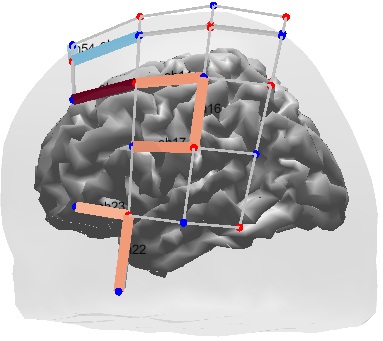 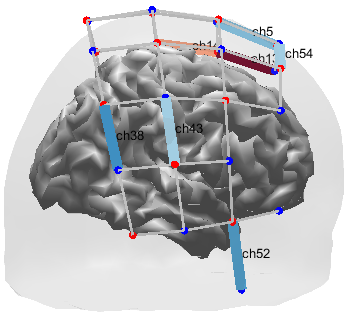 | 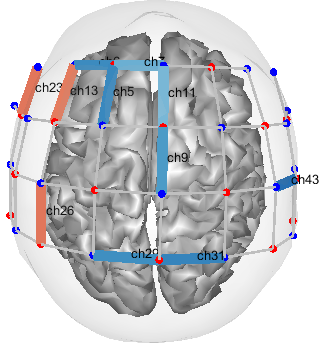  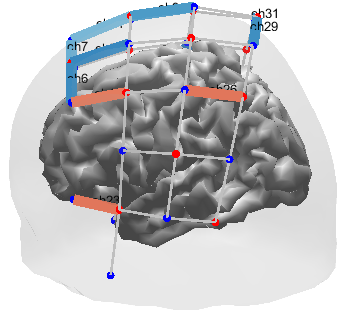 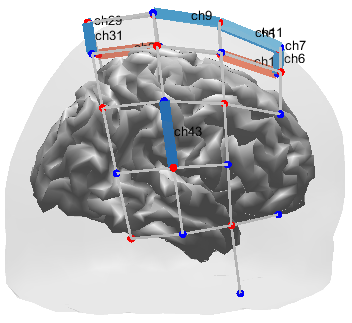 | 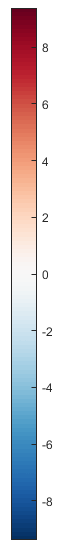 |
| **Syncopated Pacing > Synchronized Pacing** | **Syncopated Continuation > Synchronized Continuation** |  |
| 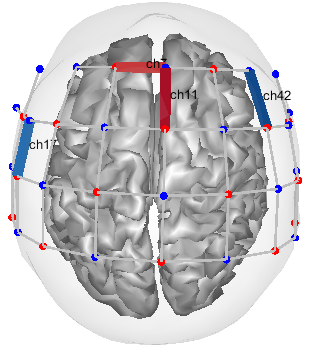  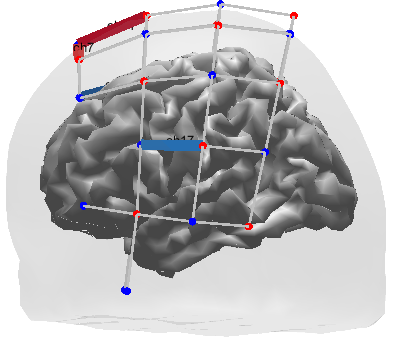 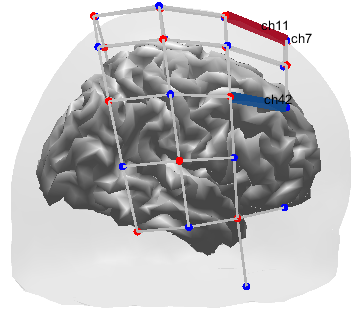 | 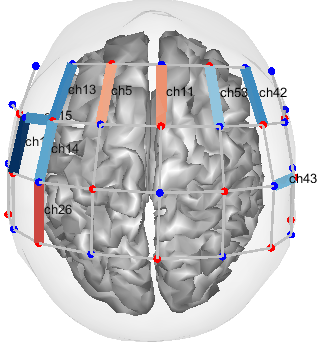  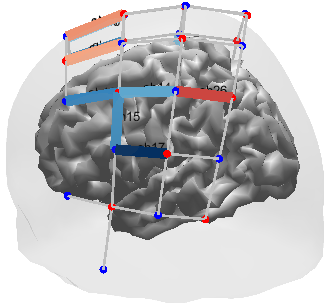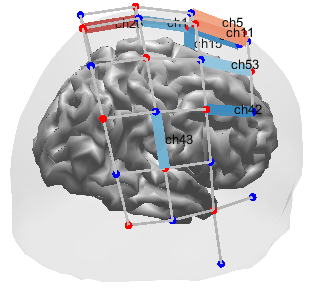 | 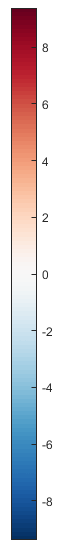 |

Figure S7. [oxy-Hb] channel maps of the contrast effect (q<0.05) obtained by block design. Color bars represent the t-value range. The channel maps were generated using the Brain AnalyzIR toolbox.

## ***Contrast maps of timing conditions in alternating design***

| **Synchronized Continuation > Synchronized Pacing** | **Syncopated Continuation > Syncopated Pacing** |  |
| --- | --- | --- |
| 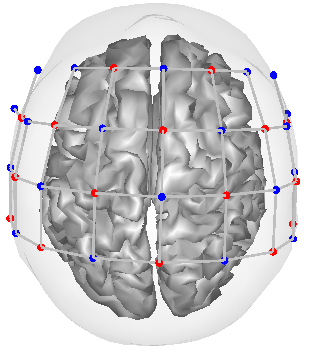  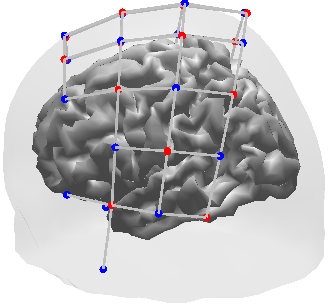 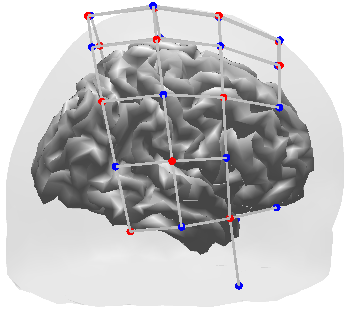 | 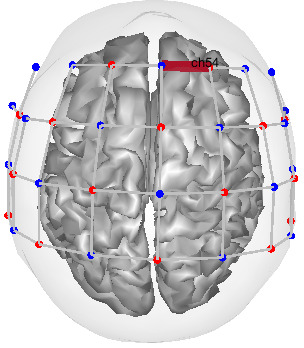  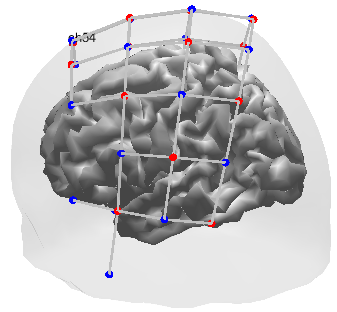 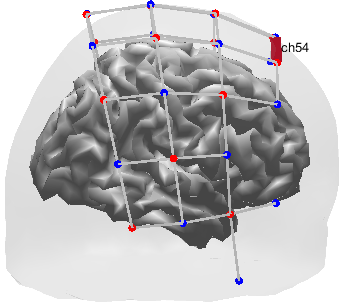 | 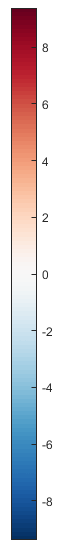 |
| **Syncopated Pacing > Synchronized Pacing** | **Syncopated Continuation > Synchronized Continuation** |  |
| 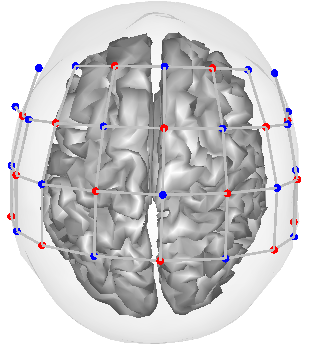  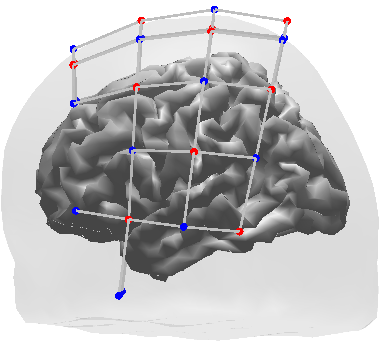 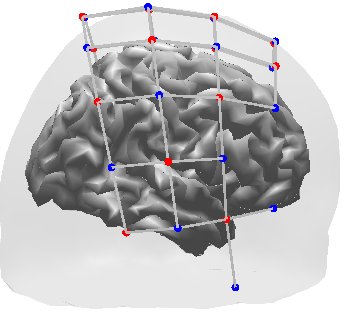 | 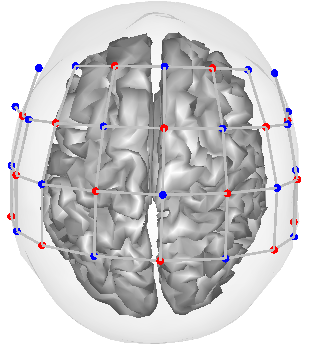  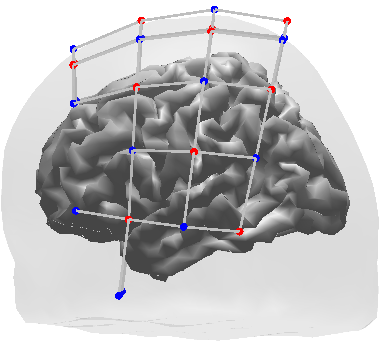 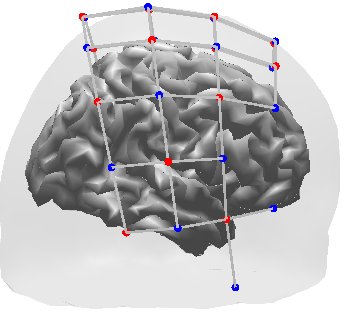 | 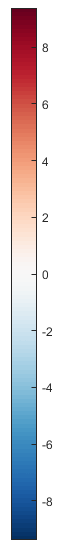 |

Figure S8. [oxy-Hb] channel maps of the contrast effect (q<0.05) obtained by alternating design. Color bars represent the t-value range. The channel maps were generated using the Brain AnalyzIR toolbox.

# **SM.4: MNI Coordinates**

Table S1. MNI coordinates according to channel numbers and source-detector pairs

| **Channel#** | **Source#** | **Detector#** | **MNI X** | **MNI Y** | **MNI Z** | **Area** |
| --- | --- | --- | --- | --- | --- | --- |
| 1 | 1 | 1 | -24 | -1 | 64 | Frontal Mid L |
| 2 | 1 | 3 | -33 | -13 | 60 | Precentral L |
| 3 | 1 | 24 | -17 | -28 | 62 | Postcentral L |
| 4 | 1 | 9 | -13 | -15 | -74 | Paracentral Lobule L |
| 5 | 2 | 1 | -19 | 32 | 59 | Frontal Mid L |
| 6 | 2 | 2 | -11 | 33 | 33 | Frontal Sup L |
| 7 | 2 | 11 | -9 | 48 | 56 | Frontal Sup L |
| 8 | 3 | 1 | -9 | 16 | 60 | Frontal Sup L |
| 9 | 3 | 9 | 0 | 4 | 81 | Supp Motor Area L |
| 10 | 3 | 10 | 14 | 22 | 71 | Supp Motor Area R |
| 11 | 3 | 11 | 2 | 32 | 67 | Frontal Sup Medial L |
| 12 | 4 | 1 | -37 | 18 | 59 | Frontal Mid L |
| 13 | 4 | 2 | -38 | 26 | 35 | Frontal Mid L |
| 14 | 4 | 3 | -32 | -2 | 43 | Precentral L |
| 15 | 4 | 4 | -39 | 12 | 27 | Frontal Inf Oper L |
| 16 | 5 | 3 | -59 | -12 | -42 | Postcentral L |
| 17 | 5 | 4 | -38 | 6 | 23 | Frontal Inf Oper L |
| 18 | 5 | 20 | -44 | -25 | 26 | Supramarginal L |
| 19 | 5 | 21 | -57 | -14 | 9 | Temporal Sup L |
| 20 | 6 | 4 | -46 | 23 | -3 | Frontal Inf Oper L |
| 21 | 6 | 21 | -52 | -3 | -6 | Temporal Mid L |
| 22 | 6 | 22 | -43 | 2 | -20 | Temporal Mid L |
| 23 | 6 | 23 | -46 | -23 | -3 | Frontal Inf Orb L |
| 24 | 7 | 20 | -54 | -40 | 11 | Temporal Sup L |
| 25 | 7 | 21 | -49 | -27 | -7 | Temporal Mid L |
| 26 | 14 | 3 | -42 | -27 | 47 | Parietal Inf L |
| 27 | 14 | 20 | -40 | -42 | 37 | Parietal Inf L |
| 28 | 14 | 24 | -32 | -40 | 57 | Parietal Inf L |
| 29 | 9 | 24 | -12 | -43 | 78 | Postcentral L |
| 30 | 9 | 9 | 0 | -31 | 73 | Paracentral Lobule R |
| 31 | 9 | 13 | 18 | -47 | 77 | Parietal Sup R |
| 32 | 10 | 9 | 15 | -18 | 66 | Supp Motor Area R |
| 33 | 10 | 10 | 34 | 4 | 73 | Frontal Sup R |
| 34 | 10 | 12 | 34 | -16 | 50 | Precentral R |
| 35 | 10 | 13 | 32 | -31 | 75 | Postcentral R |
| 36 | 11 | 12 | 36 | -25 | 46 | Postcentral R |
| 37 | 11 | 13 | 32 | -42 | 51 | Parietal Inf R |
| 38 | 11 | 14 | 46 | -42 | 39 | Supramarginal R |
| 39 | 12 | 10 | 43 | 20 | 60 | Frontal Mid R |
| 40 | 12 | 12 | 58 | 2 | 53 | Precentral R |
| 41 | 12 | 15 | 63 | 19 | 37 | Frontal Inf Oper R |
| 42 | 12 | 17 | 45 | 24 | 36 | Frontal Inf Oper R |
| 43 | 13 | 12 | 51 | -12 | 41 | Postcentral R |
| 44 | 13 | 14 | 69 | -26 | 25 | Temporal Sup R |
| 45 | 13 | 15 | 50 | 5 | 22 | Frontal Inf Oper R |
| 46 | 13 | 16 | 62 | -1 | 8 | Temporal Sup R |
| 47 | 8 | 14 | 47 | -39 | 10 | Temporal Mid R |
| 48 | 8 | 16 | 53 | -26 | -8 | Temporal Mid R |
| 49 | 15 | 15 | 48 | 9 | 10 | Rolandic Oper R |
| 50 | 15 | 16 | 51 | 0 | -4 | Insula R |
| 51 | 15 | 18 | 56 | 22 | -3 | Frontal Inf Orb R |
| 52 | 15 | 19 | 52 | 5 | -18 | Temporal Mid R |
| 53 | 16 | 10 | 23 | 31 | 55 | Frontal Sup R |
| 54 | 16 | 11 | 14 | 48 | 55 | Frontal Sup R |
| 55 | 16 | 17 | 29 | 44 | 41 | Frontal Sup R |
